# Selective fragmentation of the *trans*-Golgi apparatus by *Rickettsia rickettsii*

**DOI:** 10.1371/journal.ppat.1008582

**Published:** 2020-05-18

**Authors:** Karin Aistleitner, Tina Clark, Cheryl Dooley, Ted Hackstadt

**Affiliations:** Host-Parasite Interactions Section, Laboratory of Bacteriology, Rocky Mountain Laboratories, NIAID, NIH, Hamilton, Montana, United States of America; University of Virginia School of Medicine, UNITED STATES

## Abstract

Fragmentation of the Golgi apparatus is observed during a number of physiological processes including mitosis and apoptosis, but also occurs in pathological states such as neurodegenerative diseases and some infectious diseases. Here we show that highly virulent strains of *Rickettsia rickettsii*, the causative agent of Rocky Mountain spotted fever, induce selective fragmentation of the *trans*-Golgi network (TGN) soon after infection of host cells by secretion of the effector protein Rickettsial Ankyrin Repeat Protein 2 (RARP2). Remarkably, this fragmentation is pronounced for the *trans*-Golgi network but the *cis*-Golgi remains largely intact and appropriately localized. Thus *R*. *rickettsii* targets specifically the TGN and not the entire Golgi apparatus. Dispersal of the TGN is mediated by the secreted effector protein RARP2, a recently identified type IV secreted effector that is a member of the clan CD cysteine proteases. Site-directed mutagenesis of a predicted cysteine protease active site in RARP2 prevents TGN disruption. General protein transport to the cell surface is severely impacted in cells infected with virulent strains of *R*. *rickettsii*. These findings suggest a novel manipulation of cellular organization by an obligate intracellular bacterium to determine interactions with the host cell.

## Author summary

Intracellular pathogens have evolved multiple mechanisms to modify the host cell to promote pathogen evasion of host defense mechanisms and enhance pathogen replication. *Rickettsia rickettsii*, the agent of Rocky Mountain spotted fever, secretes a protease into the host cytosol to cause a fragmentation of the *trans*-Golgi network (TGN) with minimal effects on the *cis*-Golgi. Dispersal of the TGN disrupts trafficking of host cellular proteins to the plasma membrane. This includes inhibition of Major Histocompatibility Complex class I molecules to the cell surface thus providing *R*. *rickettsii* with a potential means of evading host immune surveillance.

## Introduction

*Rickettsia* are Gram-negative, obligate intracellular bacteria that are transmitted to humans by arthropod vectors. *Rickettsia rickettsii* is the tick-borne, causative agent of Rocky Mountain spotted fever. Since its earliest recognition, virulence of *R*. *rickettsii* has been known to be highly variable and ranges from highly virulent to avirulent strains [1, 2]. Genomic comparisons of *R*. *rickettsii* strains differing in virulence have identified a relatively limited number of mutations identifying putative virulence factors [1, 3]. Among the genes uniquely different in the avirulent Iowa strain is the Rickettsial Ankyrin Repeat Protein 2 (RARP2), recently described as a type IV secreted effector protein that associates with the ER [4]. RARP2 from *R*. *rickettsii* Iowa is truncated relative to the highly virulent Sheila Smith strain with an internal deletion of seven of the ten ankyrin repeat units observed in Sheila Smith.

Expression of *RarP2* from the virulent Sheila Smith strain (SS-RARP2) in the avirulent Iowa strain causes a change in plaque phenotype from a non-lytic to lytic phenotype mimicking that of the wild-type Sheila Smith [4]. RARP2 is a predicted clan CD cysteine protease related to eukaryotic legumains and caspases and bacterial gingipains and clostripains. Mutation of a predicted active site cysteine to alanine (C109A) reversed the effect on plaque phenotype. The additional ankyrin repeat units on SS-RARP2 are also essential for either correct targeting or increased affinity for the ER and introduction of the lytic plaque phenotype [4].

Here we show that virulent *R*. *rickettsii* strains induce selective fragmentation of the *trans*-Golgi apparatus and that this activity is mediated by RARP2. Fragmentation occurs early in the infection process, perturbs glycosylation, and disrupts transport of host cell proteins to the cell surface.

## Results

### Virulent *R*. *rickettsii* cause selective dispersal of the *trans*-Golgi apparatus

**Fig 1:**
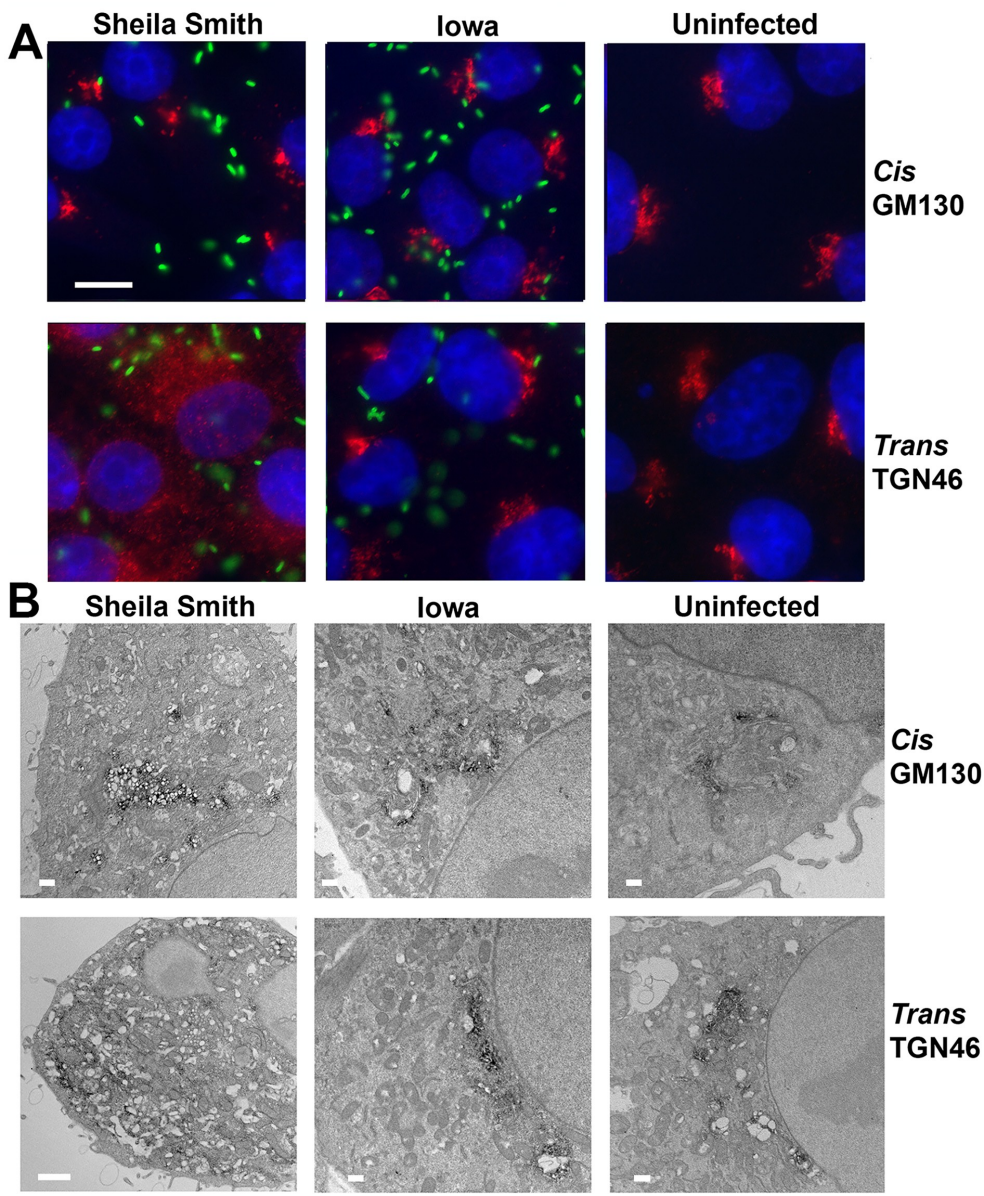
Infection with *R*. *rickettsii* Sheila Smith causes dispersal of the *trans*-Golgi network. A) Representative images showing the *trans*-Golgi network is dispersed in cells infected with the highly virulent Sheila Smith strain of *R*. *rickettsii* but not in cells infected with the avirulent Iowa strain or uninfected control cells. The *cis*-Golgi apparatus remains largely intact in cells infected with either strain. Vero cells were infected at an MOI of 1 and fixed at 24 hpi. The *cis*-Golgi protein GM130 (red) and the *trans*-Golgi protein TGN46 (red) were stained with specific antibodies. Rickettsia are labeled with anti-rOmpB specific monoclonal antibodies (green). Nucleic acids were stained with DAPI (blue). Bar = 10 μm. B) Immunoelectron microscopy confirms dispersal of the *trans*-Golgi network in cells infected with *R*. *rickettsii* Sheila Smith but not Iowa. The *cis*-Golgi apparatus appears vacuolated in cells infected with *R*. *rickettsii* Sheila Smith in contrast to cells infected with *R*. *rickettsii* Iowa or uninfected cells. Vero cells were infected at an MOI of 1 and fixed at 48 hpi. Primary antibodies targeted GM130 or TGN46, followed by horseradish peroxidase conjugated secondary antibodies and diaminobenzidine-based detection. Bar = 1 μm.

We compared the architecture of the Golgi apparatus in cells infected with *R*. *rickettsii* strains differing in virulence and found that they affected the structure of this organelle to very different degrees. Localization of the *cis*-Golgi protein GM130 revealed characteristic perinuclear Golgi-like localization in uninfected and *R*. *rickettsii*-infected host cells (Fig 1A). In contrast, the signal for the *trans*-Golgi protein TGN46 was completely dispersed in cells infected with the highly virulent Sheila Smith strain, while it was compact and resembled the uninfected controls or cells infected with the avirulent Iowa strain. These findings were corroborated by analysis of additional *trans*-Golgi specific proteins (S1 Fig).

Dispersal of the *trans*-Golgi network (TGN) by *R*. *rickettsii* Sheila Smith was verified on the ultrastructural level by immuno-electron microscopy, which showed the *trans*-Golgi protein TGN46 was dispersed throughout the cells in cells infected with the virulent Sheila Smith strain, while the *cis*-Golgi stacks labeled by GM130 were vacuolated but appropriately localized. In contrast, labeling of uninfected cells or cells infected with the avirulent Iowa strain with antibodies targeting GM130 or TGN46 showed distinct Golgi-stacks (Fig 1B, S2 Fig).

**Fig 2:**
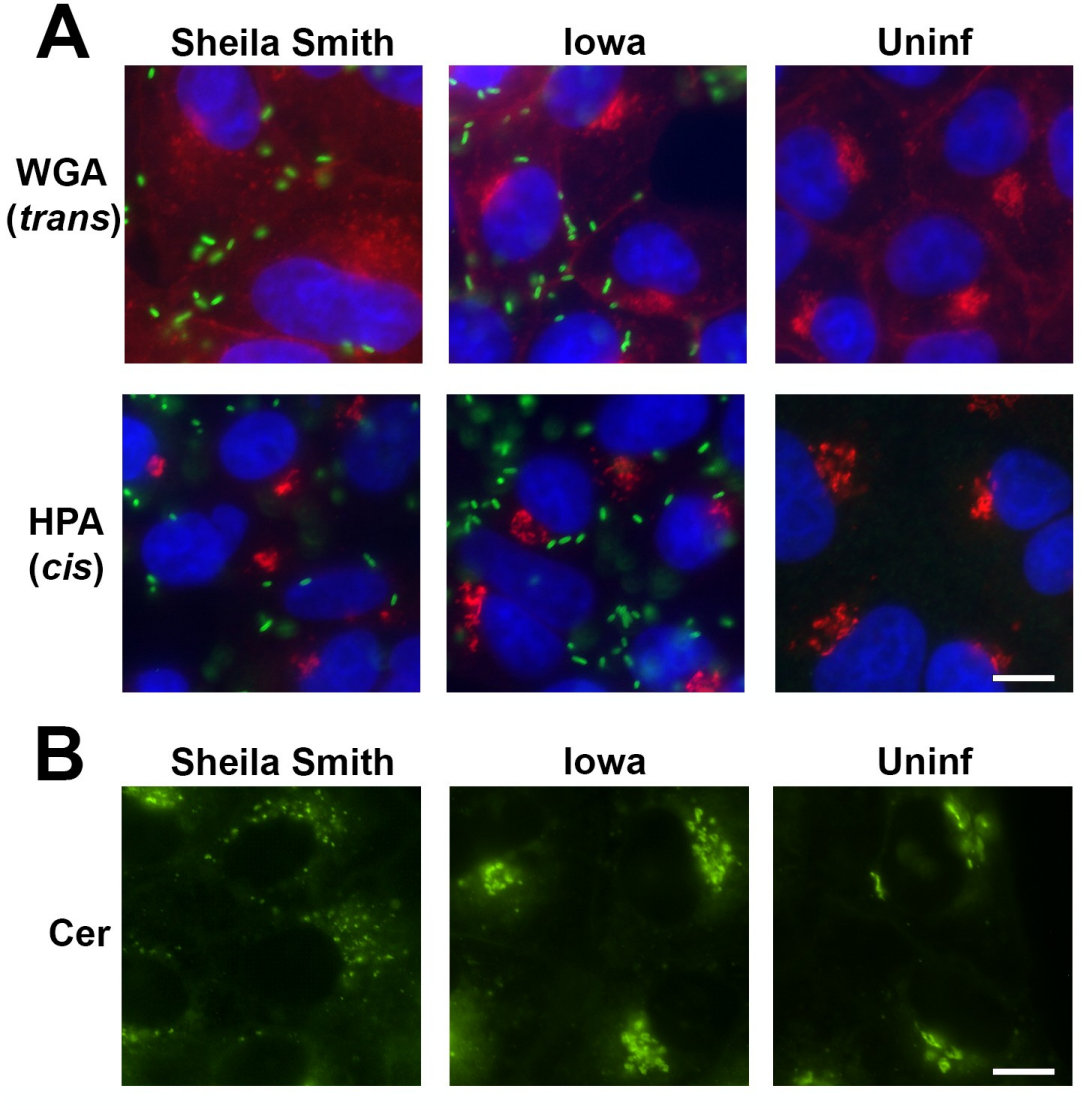
A) Lectin labeling of the Golgi apparatus in uninfected Vero cells (Uninf) or infected with *R*. *rickettsii* (green) strains Sheila Smith or Iowa for 24 hr. To confirm dispersal of the TGN using an antibody-independent labeling method, we employed the lectins wheat germ agglutinin (WGA, red) and *Helix pomatia* agglutinin (HPA; red). HPA selectively binds to terminal α-N-acetylgalactosaminyl residues–intermediate sugars added to serine and threonine residues in cis-Golgi cisternae. WGA binds sialic acid and N-acetylglucosaminyl residues and labels predominantly mature glycoproteins in the TGN [6,7]. Labeling with fluorescently labeled HPA resulted in condensed signals resembling those observed for GM130 while labeling for WGA was dispersed when cells were infected with *R*.*rickettsii* Sheila Smith. Bar = 10 um. B) Labeling of the Golgi apparatus with fluorescent C6-NBD-ceramide showed dispersed signal in cells infected with *R*. *rickettsii* Sheila Smith, but not for uninfected or *R*. *rickettsii* Iowa infected cells. Bar = 10 um.

To confirm this dispersal by an antibody-independent labeling method, we employed the labeling of the Golgi apparatus by the lectins wheat germ agglutinin (WGA) and *Helix pomatia* agglutinin (HPA). HPA selectively binds to terminal α-N-acetylgalactosaminyl residues; intermediate sugars added to serine and threonine residues in *cis*-Golgi cisternae. WGA binds sialic acid and *N*-acetylglucosaminyl residues and labels predominantly mature glycoproteins in the TGN [5–7]. Labeling with fluorescently labeled HPA resulted in condensed signals resembling signals obtained for GM130 while labeling with WGA showed dispersal of the TGN in cells infected with *R*. *rickettsii* Sheila Smith (Fig 2A).

Similarly, labeling of the Golgi apparatus with the vital stain C_6_-NBD-ceramide showed dispersed signal in cells infected with *R*. *rickettsii* Sheila Smith, but not for uninfected or *R*. *rickettsii* Iowa infected cells (Fig 2B).

### TGN fragmentation is due to the secreted effector protein RARP2

**Fig 3:**
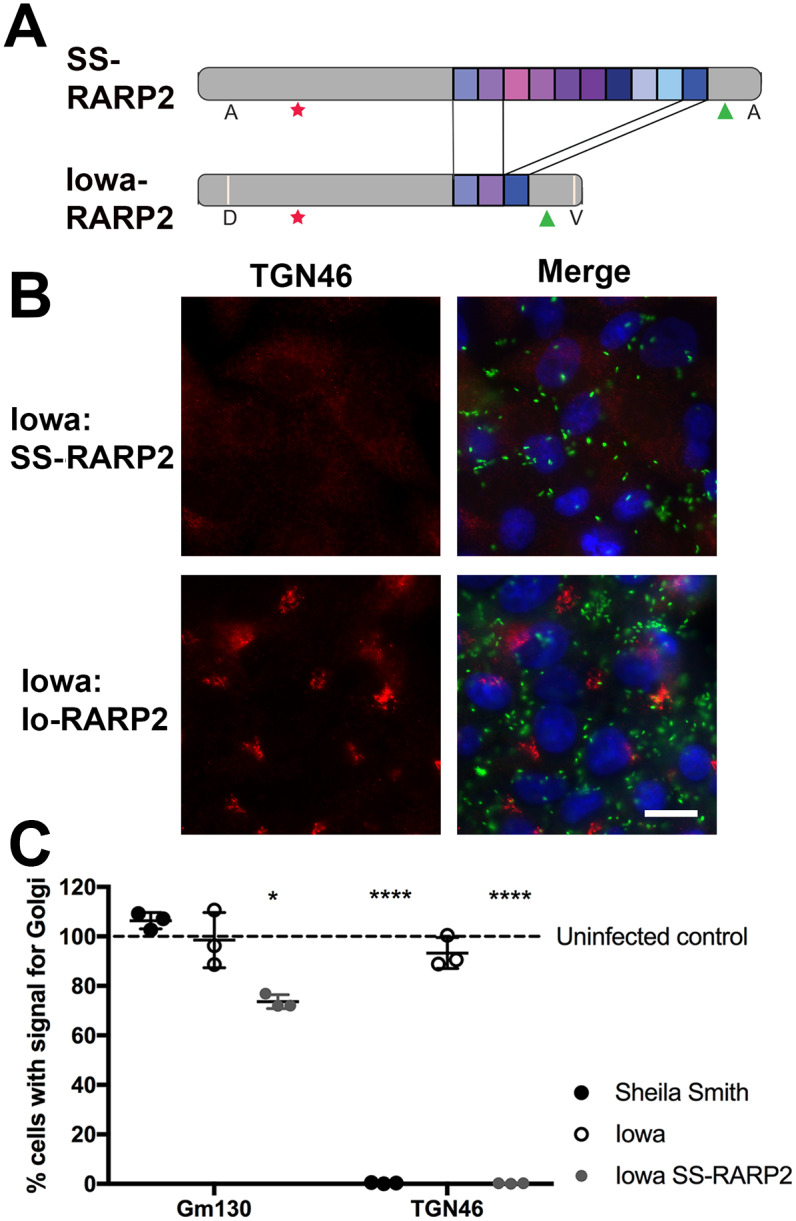
The effector protein RARP2 of *R*. *rickettsii* Sheila Smith causes dispersal of the *trans*-Golgi network. A) Schematic of RARP2 variants showing *R*. *rickettsii* Sheila Smith (SS-RARP2) contains 10 ankyrin repeats units relative to RARP2 of *R*. *rickettsii* Iowa (Iowa-RARP2) which contains only 3 Ankyrin repeats. The cleavage site of the type IV secretion signal (green triangle), a predicted catalytic cysteine (red asterisk) and non-synonymous SNPs (white bar in the Iowa protein schematic) are identified. B) Expression of RARP2 of *R*. *rickettsii* Sheila Smith (Iowa:SS-RARP2), but not RARP2 of the Iowa strain (Iowa:Io-RARP2) in the avirulent Iowa strain causes dispersal of the *trans*-Golgi network. Genes were cloned into the vector pRAMF2, transformed into *R*. *rickettsii* Iowa and used to infect Vero cells. Cells were fixed 24 hpi and the *trans*-Golgi protein TGN46 (red) and rickettsiae (green) were detected by specific antibodies. Nucleic acids were stained with DAPI (blue). Bar = 10μm. C) Quantitation of the TGN46 observed in cells infected with *R*. *rickettsii* Sheila Smith or Iowa or infected with *R*. *rickettsii* Iowa expressing SS-RARP2. GM130 and TGN46 localization was determined for at least 350 host cells per strain for each of three biological replicates and normalized to uninfected control cells. Shown is the mean +/- the S.E.M. Statistics were performed using One-way ANOVA and post-hoc Tukey test. Significant differences to the uninfected control are indicated (**** p<0.0001 and * p < 0.05).

Genomic comparisons of *R*. *rickettsii* strains differing in virulence have identified a relatively limited number of mutations representing putative virulence factors [1, 3]. One of the few genes that differ between virulent *R*. *rickettsii* strains and the avirulent Iowa strain is the Ankyrin-repeat protein RARP2, an effector protein that has been previously shown to be secreted into the host cell [1, 3, 4]. RARP2 of the Iowa (Io-RARP2) strain and the moderately virulent R strain contain only three Ankyrin repeat units vs. ten Ankyrin repeats in RARP2 of the Sheila Smith (SS-RARP2) and Morgan strains (Fig 3A) [4]. We therefore compared the effects of these additional strains of *R*. *rickettsii* on the structure of the Golgi apparatus (S3 Fig). Both the Sheila Smith and the Morgan strains caused dispersal of the *trans*-Golgi but the *cis*-Golgi remained intact. In contrast, the two strains with a truncated RARP2, the Iowa and R strains, did not show morphological evidence for disruption of the TGN. Two members of the Typhus group of rickettsiae, *R*. *typhi* and *R*. *canadensis*, do not show disruption of the TGN (S4 Fig).

To confirm that SS-RARP2 plays a role in dispersal of the TGN, we infected Vero cells with *R*. *rickettsii* Iowa transformed with the rickettsial expression plasmid pRAMF2 encoding SS-RARP2. Expression of the Sheila Smith RARP2 from the Iowa strain resulted in the dispersal of the TGN and condensed signals for the *cis*-Golgi protein GM130 as observed after infection of cells with the virulent Sheila Smith strain (Fig 3B). Dispersal of the TGN46 signal was observed in nearly 100% of cells infected with either Sheila Smith or Iowa expressing SS-RARP2, while the parental Iowa strain showed TGN46 labeling similar to the uninfected controls (Fig 3C). Expression of pRAMF2-encoded Io-RARP2 from the Iowa strain did not cause dispersal of the *trans*-Golgi network, indicating that dispersal is not the result of overexpression of RARP2 (Fig 3B). SS-RARP2, but not Io-RARP2, also caused dispersal of the TGN when *R*. *montanensis*, an avirulent species not encoding a RARP2-homolog, was transformed with the corresponding pRAMF2 constructs (S5 Fig).

### The proteolytic activity of RARP2 is necessary for TGN fragmentation

**Fig 4:**
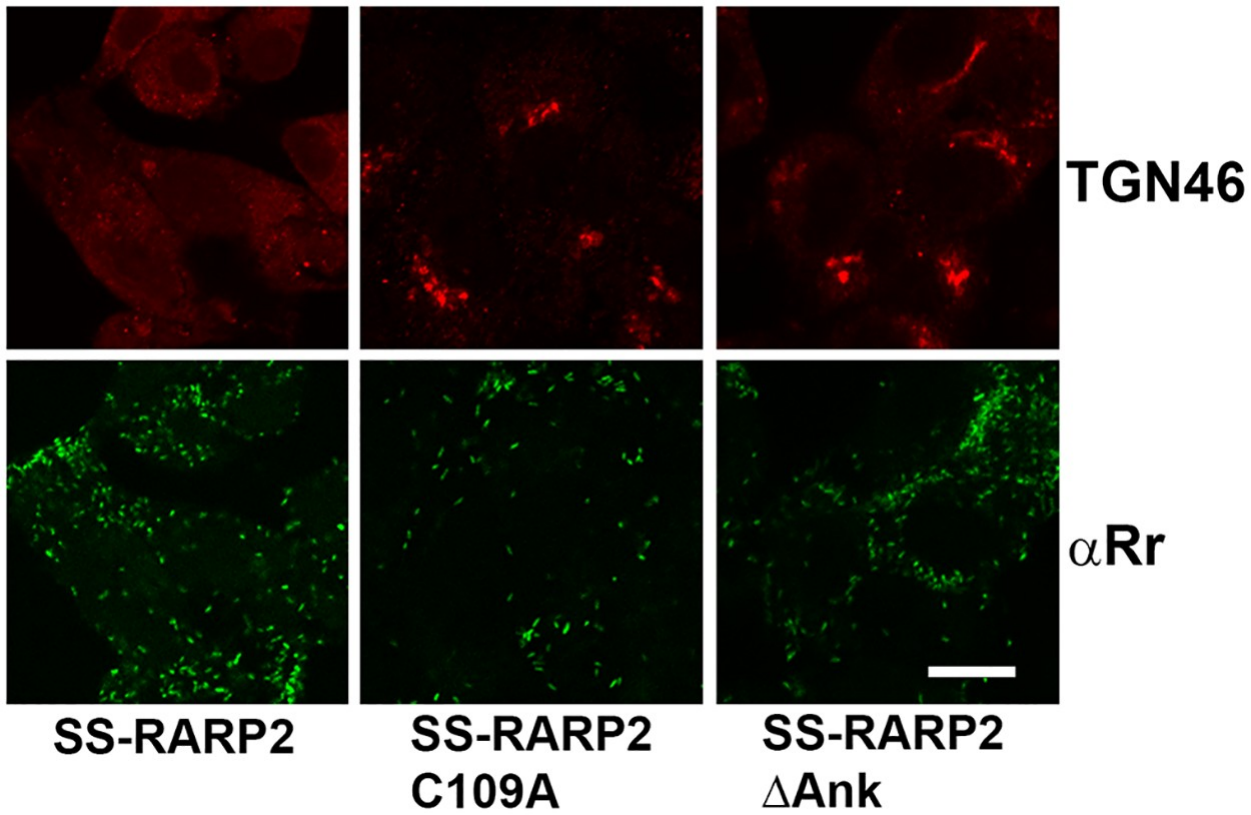
Mutation of a putative catalytic cysteine residue or deletion of the ankyrin-repeat domain abolishes TGN dispersal by SS-RARP2. *R*. *rickettsii* Iowa expressing SS-RARP2 carrying a mutation of the putative catalytic cysteine at position 109 to an alanine (SS-RARP2-C109A); *R*. *rickettsii* Iowa expressing SS-RARP2 with the ankyrin-repeat domain deleted (SS-RARP2ΔAnk); *R*. *rickettsii* Iowa expressing SS-RARP2 (SS-RARP2) were used to infect Vero cells. Cultures were fixed at 48 hpi and stained for TGN46 (red) and *R*. *rickettsii* (green). Bar = 10 μm.

RARP2 is predicted to contain a structural domain with similarities to CD clan cysteine proteases [4]. Expression of SS-RARP2 in R. rickettsii Iowa causes a change to a lytic plaque phenotype typical of Sheila Smith. Mutation of a putative catalytic cysteine site to an alanine in the N-terminus of SS-RARP2 (C109A SS-RARP2) maintains the native non-lytic plaque phenotype of the Iowa strain [4]. Consistent with these earlier observations, *R*. *rickettsii* Iowa expressing the C109A SS-RARP2 mutant failed to induce dispersal of the TGN (Fig 4). Similarly, deletion of the ankyrin repeats in the SS-RARP2 construct abolished dispersal of the TGN in host cells. This demonstrates that both the ankyrin repeats and the putative catalytic cysteine are required for disruption of the *trans*-Golgi network by SS-RARP2 and suggests that proteolytic activity is necessary for RARP2 function.

**Fig 5:**
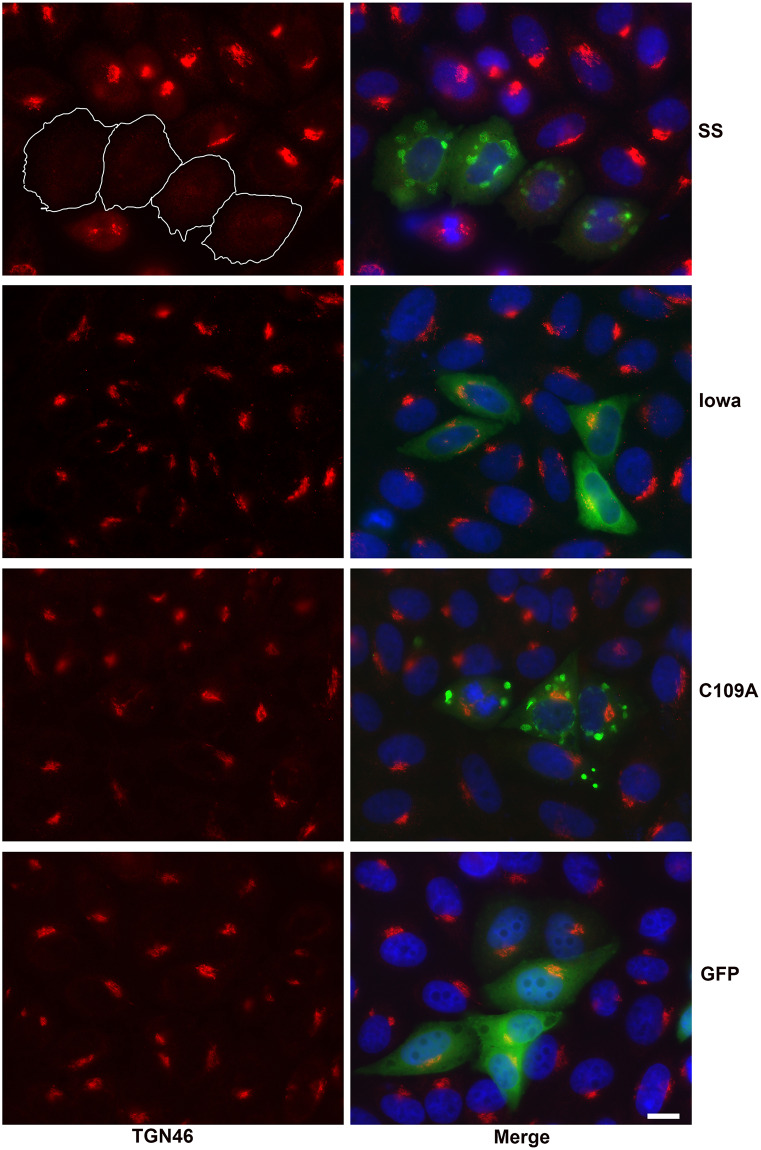
Ectopic expression of SS-RARP2 causes dispersal of the *trans*-Golgi network. Sheila Smith RARP2 (SS), Iowa RARP2 (Iowa), or the SS-RARP2-C109A mutant (C109A) were expressed in Vero cells as EGFP fusions from pEGFP-C1 and probed for TGN46 (red). Parental pEGFP-C1 transfected cells served as a negative control. The TGN is dispersed in cells expressing SS-RARP2 (green), but not in cells expressing Iowa-RARP2, SS-RARP2-C109A, or negative control cells expressing EGFP (green). Ectopically expressed SS-RARP2-GFP and SS-RARP2-C109A are enriched in vesicular structures as previously observed [4]. Outlines of transfected cells expressing SS-RARP2 are shown in white. Bar = 10 μm. Higher magnification images are shown in S6 Fig.

To verify the effects of SS-RARP2 and Io-RARP2 on mammalian cells in the absence of other rickettsial proteins, we transfected Vero cells with Sheila Smith RARP2, Iowa RARP2, or the SS-RARP2-C109A mutant expressed as EGFP fusions from pEGFP-C1 and probed for Golgi structure (Fig 5). The TGN is dispersed only in cells expressing SS-RARP2, but not in cells expressing Iowa-RARP2, SS-RARP2-C109A, or negative control cells expressing EGFP. A higher magnification image is shown in S6A Fig. Ectopically expressed SS-RARP2-GFP and SS-RARP2-C109A are enriched in vesicular structures as previously observed [4]. Quantitation of the dispersal of the TGN in SS-RARP2 transfected cells confirmed that the TGN disruption was highly significant (p < .0001) when compared to cells transfected with Iowa-RARP2-GFP, SS-RARP2-C109A-GFP, or EGFP (S6B Fig).

### Dispersal of the *trans*-Golgi network occurs early in infection and requires rickettsial protein expression

**Fig 6:**
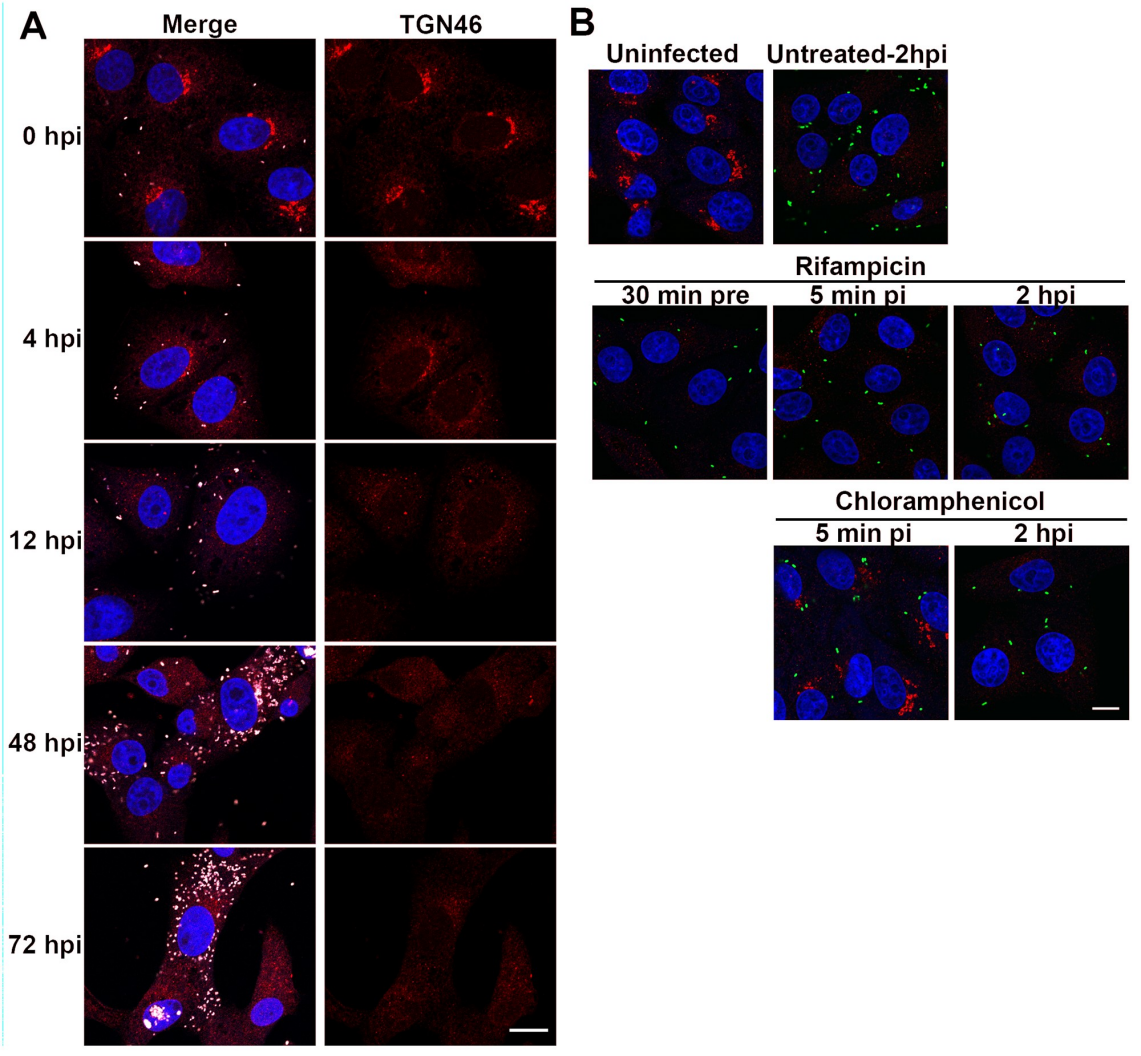
Temporal analysis of TGN dispersal. A) Time course of *trans*-Golgi dispersal in *R*. *rickettsii* Sheila Smith infected cells. Infected cells were fixed and labeled at the indicated times. TGN disruption is evident by 4 hpi. TGN46 (red), rickettsiae (pink), and DAPI (blue). Bar = 10 um. B) Effects of inhibitors of rickettsial transcription (rifampicin) or translation (chloramphenicol) when added at various times pre- or post-infection. Inhibitors were added at 30 min pre-infection (30 min pre), 5 min post-infection (5 min pi), or 2 hr post-infection (2 hpi). Cultures were fixed and stained at 24 hpi. Uninfected cells are shown in the upper right of panel B. The remainder of the cells are infected with *R*. *rickettsii* Sheila Smith (green). TGN46 is shown in red and cells couterstained with DAPI to identify the nucleus. Bar = 10 μm.

Following uptake by the host cell, *Rickettsia* escape the phagocytic vacuole rapidly and initiate replication in the host cell cytoplasm. The TGN is dispersed by 4 hpi in cells infected with *R*. *rickettsii* Sheila Smith, but not *R*. *rickettsii* Iowa. By 72 hpi many of host cells had lysed when cells were infected with the virulent Sheila Smith strain, but the remaining cells still showed dispersal of TGN46 (Fig 6A). Disruption of the TGN thus occurs early during infection when very low numbers of rickettsiae are present.

To test whether rickettsial transcription and translation are necessary for this early dispersal of the TGN, we inhibited both processes by the addition of rifampicin or chloramphenicol, respectively, to infected cells at early times pre- and post-infection. Effects on Golgi structure were examined at 24 hpi. The transcriptional inhibitor rifampicin prevented growth of rickettsiae, but not dispersal of the TGN, even if rickettsiae were preincubated with rifampicin before infection (Fig 6B). Addition of chloramphenicol prevented dispersal of the TGN if the antibiotic was added to the cells immediately after infection, but not if it was added at 2 hpi or later timepoints. This suggests that the mRNA encoding for the rickettsial protein causing TGN dispersal is present in rickettsiae before infection, and that the translation of the protein and initiation of TGN dispersal occurs very early in infection.

To determine whether TGN dispersal is reversible, chloramphenicol was added to *R*. *rickettsi*-infected cells at 24 hpi to inhibit bacterial protein synthesis. Addition of chloramphenicol resulted in the restoration of Golgi morphology following an additional 24 hr incubation while untreated cells retained the dispersed Golgi architecture (S7 Fig).

### Glycosylation defect in *R*. *rickettsii* Sheila Smith infected cells

**Fig 7:**
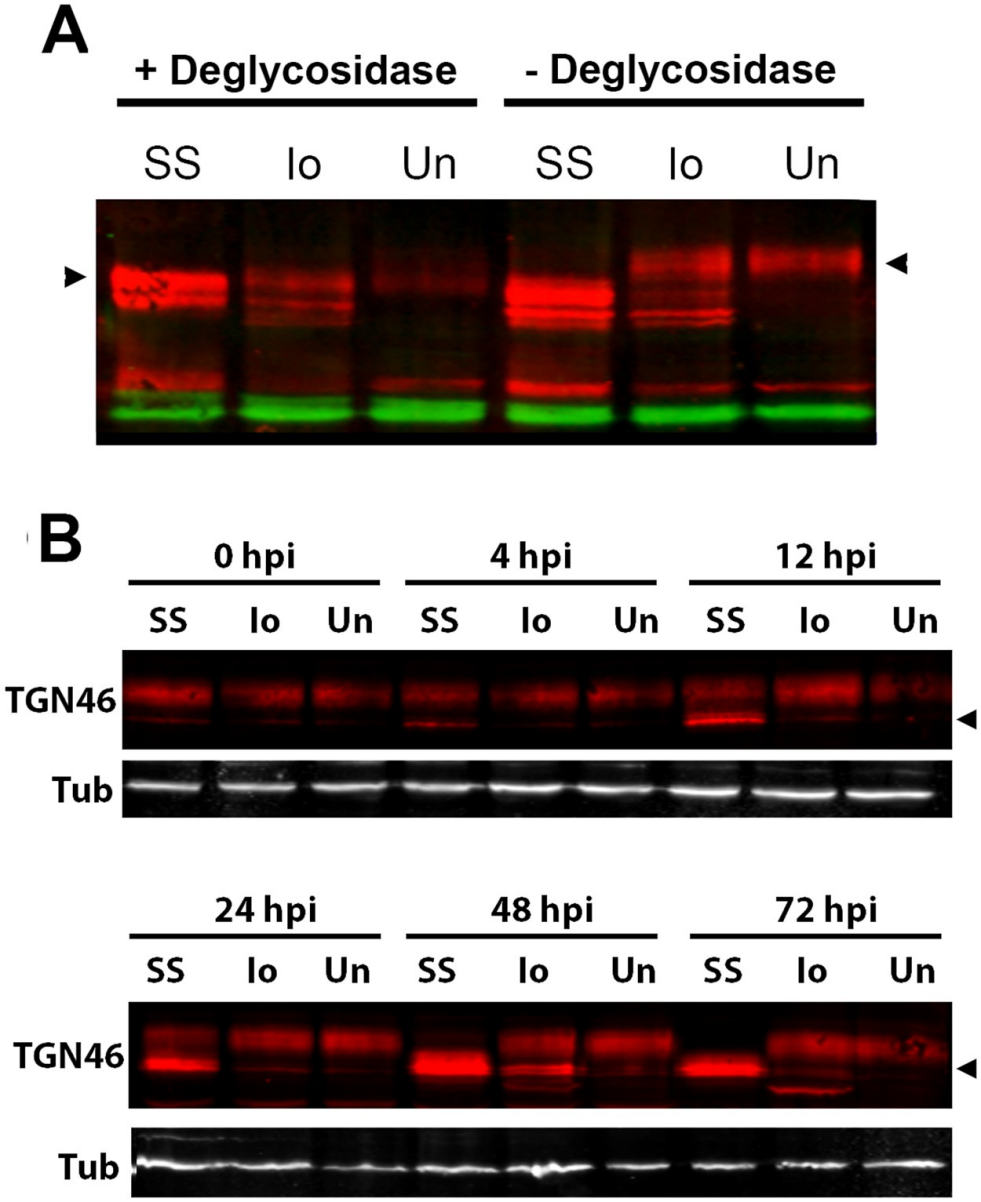
TGN46 glycosylation defects in Sheila Smith infected cells. A) TGN46 in *R*. *rickettsii* Sheila Smith (SS) infected cells is unglycosylated and therefore shows no effect of deglycosylation treatment. *R*. *rickettsii* Iowa (Io) or uninfected Vero cell lysates (Un) migrate at a lower apparent mol. wgt. following sialidase treatment. Red represents TGN46; tubulin (green) was used as a loading control. Left arrowhead indicates the major deglysylated form of TGN46; right arrowhead the glycosylated form. B) Temporal analysis of TGN46 deglycosylation. Uninfected Vero cells (Un) or cells infected with *R*. *rickettsii* Sheila Smith (SS) or Iowa (Io) were harvested for SDS-PAGE at various times post infection and immunoblotted for TGN46, which identifies an immature glycosylated form of TGN46 (arrowhead) apparent by 4 hpi. A lesser amount of unglycosylated TGN46 is apparent even in the Iowa infected cells at the later time points at 48 and 72 hpi. We believe this represents activity of RARP2 which even though inappropriately targeted, may be in sufficient quantity by that time to show an effect. Tubulin (Tub) was used as a loading control.

Concomitant with TGN dispersal was the appearance of deglycosylated forms of TGN46, indicating a defect in protein glycosylation associated with fragmentation of the TGN in infected cells (Fig 7). Western blot analysis of the *trans*-Golgi protein TGN46 showed a shift of this protein to a lower molecular weight in cells infected with *R*. *rickettsii* Sheila Smith, compared to uninfected cells or cells infected with the Iowa strain (Fig 7A). TGN46 is glycosylated which increases its apparent molecular mass [8]. TGN46 migrated with a lower apparent mol. mass in lysates of cells infected with *R*. *rickettsii* Sheila Smith and no shift was seen after treatment to deglycosylate the protein. TGN46 migrated at a higher apparent mol. mass in uninfected cells or cells infected with the Iowa strain. Treatment of cell lysates of Iowa-infected or uninfected cells with deglycosylation mix resulted in a shift of TGN46 to a lower molecular mass due to cleavage of oligosaccharide side chains. These results suggest that dispersal of the TGN is associated with a protein glycosylation defect in cells infected with *R*. *rickettsii* Sheila Smith.

A time course experiment demonstrated the appearance of immature, unglycosylated forms of TGN 46 in Sheila Smith infected cells as early as 4 hr post-infection is consistent with the observed disruption of the Golgi apparatus at similar times post-infection (Fig 7B). These findings do not, however, distinguish whether the reduced glycosylation of TGN46 is a cause or result of TGN disruption.

### *Cis*-Golgi function is not necessary for rickettsial growth

We considered the possibility that rickettsial fragmentation of primarily the *trans*-Golgi network might imply that preservation of the *cis*-Golgi may in some way be beneficial to rickettsiae. To test this possibility, we infected Vero cells with *R*. *rickettsii* strains Sheila Smith and Iowa in the presence or absence of Brefeldin A to disrupt the Golgi apparatus and followed progeny production over 48 hrs (S8 Fig). No significant differences in growth rates were observed; indicating that, at least in cell culture, maintenance of the *cis*-Golgi is not essential for rickettsial growth.

#### Dispersal of the TGN disrupts protein trafficking to the infected cell surface

**Fig 8:**
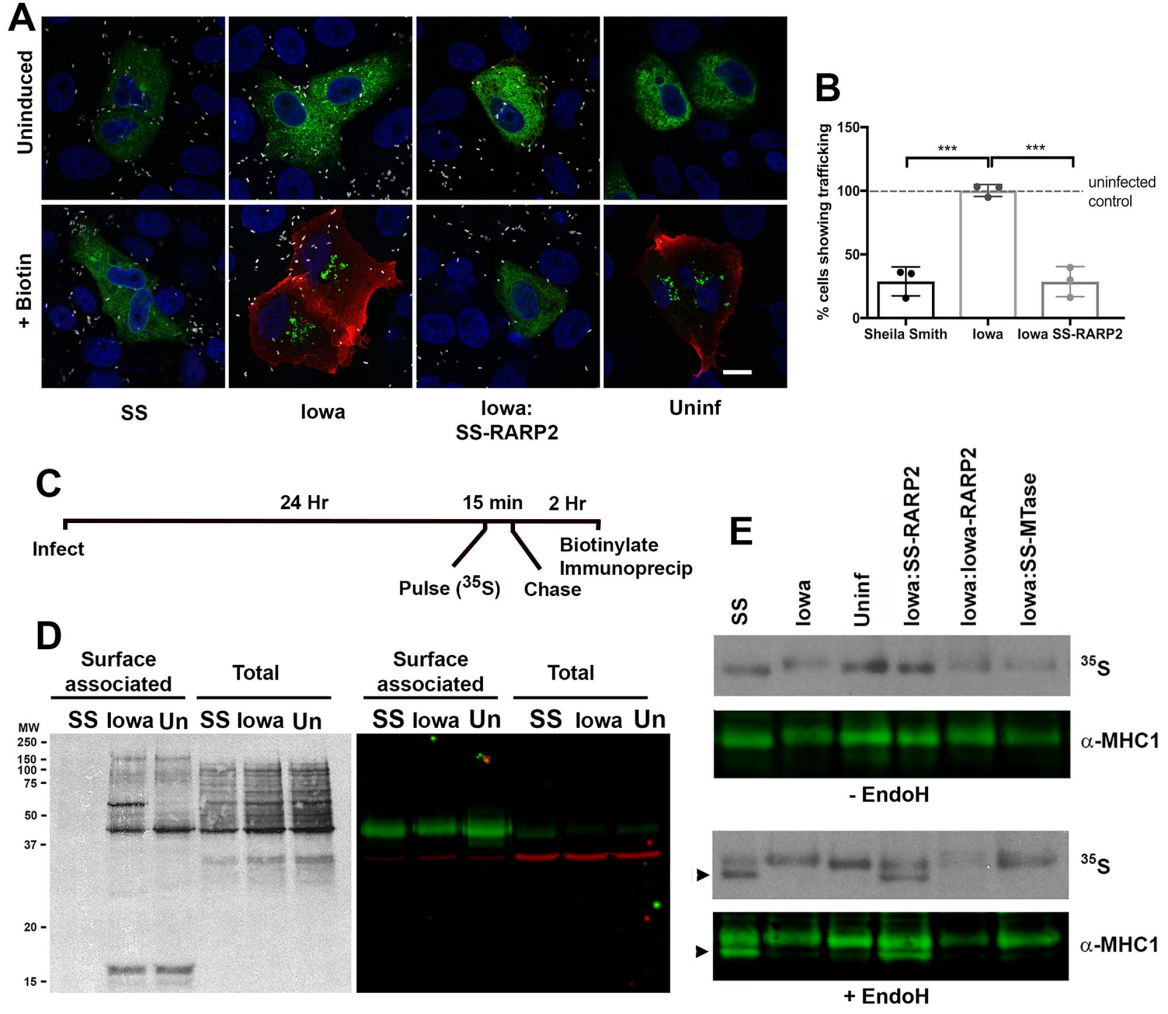
SS-RARP2 mediated dispersal of the *trans*-Golgi interferes with protein transport to the host cell surface. A) Infection of Vero cells with *R*. *rickettsii* Sheila Smith (SS) or Iowa expressing SS-RARP2 (Iowa:SS-RARP2) inhibits trafficking of GFP-tagged TNFalpha to the cell surface. Vero cells were transfected with RUSH-constructs [9] encoding GFP-tagged TNFalpha and infected with rickettsiae 24 hours after transfection. Biotin was added 24 hpi to release reporter proteins from the endoplasmic reticulum and fixed 80 min after initiation of trafficking. GFP-tagged reporter proteins on the host cell surface were detected with anti-GFP antibody (red) without permeabilization, while intracellular GFP-tagged reporter protein is shown in green. A red signal thus indicates normal trafficking to the cells surface such that the reporter is detected with the anti-GFP antibody and red secondary antibody. Nucleic acids stained with DAPI are shown in blue, rickettsiae in white. B) Quantification of cells showing surface-labeling for GFP. Signals were counted for at least 100 cells for each of three biological replicates, and numbers were normalized to uninfected control cells. Shown is the mean +/- the S.E.M. Statistics were performed using One-way ANOVA and post-hoc Tukey test. Significant differences to the uninfected control are indicated with *** (p < 0.0005). C) Vero cells were infected with rickettsiae, and pulse-labeled for 15 min. with ^35^S-methionine, incubated for two more hours, surface biotinylated and then surface proteins pulled-down with streptavidin coated beads for SDS-PAGE, autoradiography, and immunoblotting. D) Radioactively-labeled surface proteins are pulled down from uninfected cells (Un) and cells infected with the Iowa strain (Io), but surface exposure of newly synthesized protein is greatly inhibited in cells infected with *R*. *rickettsii* Sheila Smith (SS). Autoradiographs of surface-associated proteins and total cell lysates are shown in the left hand panel and demonstrate a substantial decrease in surface exposure of *de novo* synthesized proteins in *R*. *rickettsii* Sheila Smith (SS) infected cells. The Western blot on the right demonstrates that MHC-1 was pulled down under these conditions and indicates the position of MHC-I (green) in surface protein and total protein lysates. GAPDH (red) is used as a loading control and seen primarily in the total cell lysates. Molecular weight (MW) is indicated on the left. E) A large fraction of newly synthesized MHCI is sensitive to Endoglycosidase H (EndoH) treatment in cells infected with *R*. *rickettsii* Sheila Smith (SS) or Iowa expressing SS-RARP2, indicating incomplete processing and maturation of the glycoprotein in the Golgi apparatus. Cells were pulsed-labeled with ^35^S, and MHC-I was pulled down with specific antibodies. Western blots (α-MHCI) and autoradiographs (^35^S) of pulled-down proteins treated with EndoH (+ EndoH) or untreated (- EndoH) are shown. Iowa expressing Io-RARP2 and Iowa expressing a methyltransferase of the Sheila Smith strain (Iowa:SS-MTase) were used as controls. Arrowheads indicate the position of the EndoH-sensitive, immature forms of MHC-I.

We employed two independent methods to determine effects of *R*. *rickettsii*-mediated disruption of the TGN on host protein trafficking to the plasma membrane. We first used the Retention Using Selective Hooks (RUSH) system [9] to study the impact of the SS-RARP2-mediated TGN disruption on protein trafficking. In this system, proteins of interest are fused to GFP and a streptavidin binding peptide (SBP) and co-expressed with streptavidin, which is targeted to the endoplasmic reticulum. Addition of biotin displaces the SBP-reporter fusion from the ER-localized streptavidin to synchronously release the reporter for trafficking to its target compartment. 24 hours after transfection with a TNFα-SBP-GFP reporter protein, Vero cells were infected with rickettsiae, and synchronous release of the reporter protein and trafficking to the plasma membrane initiated by the addition of biotin at 24 hpi. Prior to the addition of biotin, the TNF-α-SBP-eGFP reporter protein was anchored in the endoplasmic reticulum (ER) (Fig 8A). 80 min after the addition of biotin, EGFP-reporter proteins were detected at the cell surface of unpermeabilized, uninfected cells or cells infected with *R*. *rickettsii* Iowa with an anti-GFP antibody. In contrast, the reporter proteins were not detected at the cell surface in most cells infected with *R*. *rickettsii* Sheila Smith or Iowa expressing SS-RARP2. Instead, the reporter construct was retained in the endoplasmic reticulum, demonstrating that cellular trafficking is impeded after dispersal of the TGN (Fig 8A and 8B).

These results were extended by ^35^S pulse-labeling experiments with infected and uninfected cells followed by surface biotinylation (Fig 8C). Biotinylated, radiolabeled surface proteins, representing newly-synthesized protein, were precipitated from uninfected cells and cells infected with the Iowa strain, however, newly-synthesized proteins were nearly undetectable in surface fractions of cells infected with *R*. *rickettsii* Sheila Smith (Fig 8D). Total lysates of infected and uninfected cells showed similar patterns of radioactively-labeled proteins indicating that the defect was one of trafficking to the host cell surface. Immunoblotting of surface fractions obtained by streptavidin precipitation showed similar levels of total, surface associated, major histocompability complex I molecules (MHC-I) in Western blots (Fig 8D). This demonstrates that although *de novo* synthesized MHC-1 shows reduced delivery to the cell surface, pre-existing MHC-1 remains exposed on the plasma membrane.

To substantiate a defect in MHC-I trafficking in Sheila Smith-infected cells, we monitored the acquisition of resistance to Endo H upon processing of glycoproteins in the Golgi apparatus. While the core-glycosylation of glycoproteins with N-glycans, which are acquired in the ER, is susceptible to removal by EndoH, the more complex glycosyl groups added in the mid- and *trans*-Golgi apparatus are Endo H-resistant [6]. Endo H treatment of cell lysates showed that a large fraction of newly-synthesized, radiolabeled MHC I was susceptible to cleavage in cells infected with *R*. *rickettsii* Sheila Smith or Iowa expressing SS-RARP2 (Fig 8E). Infection with *R*. *rickettsii* Iowa or the Iowa strain expressing Io-RARP2 or a rickettsial control protein (MTase) did not change levels of unprocessed MHC-I in comparison to uninfected controls (Fig 8E). Collectively, these experiments show that dispersal of the TGN caused by *R*. *rickettsii* Sheila Smith interferes with MHC-I trafficking to the cell surface, which may aid in evasion of immune surveillance.

## Discussion

Many bacterial pathogens have developed strategies to subvert and exploit organelles of eukaryotic cells [10]. Virulent strains of *R*. *rickettsii* induce fragmentation of the *trans*-Golgi apparatus soon after infection of host cells by secretion of the effector protein RARP2. RARP2 is a predicted cysteine protease [4] that induces selective fragmentation of the TGN and thereby disrupts cellular protein trafficking to the plasma membrane. The selective fragmentation of the *trans*-, but not the *cis*- Golgi apparatus, observed after infection with highly virulent strains of *R*. *rickettsii* differs from fragmentation of the Golgi apparatus caused by other physiological processes or infections. Fragmentation of the Golgi apparatus is commonly observed during a number of cellular processes such as mitosis [11] and apoptosis [12]. Fragmentation of the Golgi can also be induced by chemical inhibitors such as Brefeldin A [13], or nocodazole. Pathological processes including neurodegenerative diseases such as Alzheimer's disease result in loss of Golgi structure [14] and infection with certain viruses or intracellular bacteria can also cause Golgi dispersal [15–19]. The recognition of a bacterial effector protein that preferentially disrupts a specific Golgi compartment may help unravel the intricacies of Golgi structure and function in the future.

Few examples of bacteria fragmenting the Golgi apparatus are known. The effector proteins IpaJ, VirA or IpaB, have been implicated in fragmentation of the Golgi apparatus in *Shigella flexneri* infected cells and resultant inhibition of protein trafficking [20,21]. The obligate intracellular bacteria, *Anaplasma phagocytophilum* [22] and *Orientia tsutsugamushi* [23] also perturb Golgi architecture to differing degrees. The obligate intracellular bacterium *Chlamydia trachomatis* has been described as fragmenting the Golgi complex into ministacks [24] yet the fragmentation observed is not as extensive as that observed for *R*. *rickettsii*, nor does *C*. *trachomatis* induce defects in protein processing or trafficking [25].

The Golgi apparatus is a highly dynamic organelle. Critical to its function as the site for post-translational modification of newly synthesized proteins and sorting of traffic to different sites within the cell is its structural organization into membranous Golgi stacks. Newly synthesized proteins enter from the ER into the *cis*-Golgi. Cargo is post-translationally modified as it transits the Golgi apparatus before sorting and trafficking from the *trans*-Golgi network. During cell division, the Golgi completely disperses and re-organizes upon completion of mitosis. There are multiple cellular factors involved in establishment and maintenance of Golgi architecture. Among these are various SNARE-like proteins, Rab GTPases, coat proteins (COPI, COPII, clathrin), peripheral membrane proteins known as golgins, as well as the actin and microtubule cytoskeleton [26–30]. In addition, protein kinases and phosphatases as well as ubiquitin ligases and deubiquitinating enzymes play regulatory roles throughout these processes [31]. Intracellular pathogens that disrupt the Golgi apparatus have been found to target many of the above factors important to Golgi organization including Rab GTPases [22], Golgins [24,32], COPII [23], microtubules [15,19], activation of Src-family kinases [33], and disruption of Golgi lipid composition [21]. The cellular target of RARP2 remains unknown. The mechanism, however, almost certainly involves proteolysis of a cellular substrate since mutation of the putative active site cysteine of RARP2 prevents fragmentation of the Golgi. The ankyrin repeat domain also appears essential for appropriate targeting of the protease. As previously shown, the ankyrin repeat domain targets RARP2 to structures bearing ER markers [4]. It is possible that the cellular target is present, at least transiently, in the ER before transit to more distal sites within the Golgi. Identification of the cellular target of RARP2 remains a primary goal.

The absence of TGN dispersal in cells infected with the avirulent *R*. *rickettsii* Iowa strain is correlated with the reduced number of ankyrin-repeat units of Io-RARP2 relative to SS-RARP2 (3 vs. 10). Their importance is supported by the prevention of TGN-fragmentation if ankyrin-repeats are deleted from SS-RARP2. Similarly, RARP2 from the R strain of *R*. *rickettsii*, which shows a lesser degree of virulence than the Sheila Smith [4], contains only three ankyrin repeats units as does the Iowa strain and also fails to induce Golgi fragmentation. The number of individual repeats within ankyrin-repeat proteins is known to impact their structure and flexibility as interaction platforms [34]. RARP2 from *R*. *rickettsii* Sheila Smith was observed to associate with pleomorphic membranous structures bearing ER markers whether expressed as recombinant proteins from rickettsiae or expressed ectopically in eukaryotic cells, however, RARP2 from Iowa did not. RARP2 of either strain was shown to be expressed and secreted from rickettsiae into the host cytosol [4]. We speculated that the truncated RARP2 from Iowa might therefore fail to localize appropriately to ER membrane. The available evidence suggests that appropriate targeting to the ER is important for TGN disruption. RARP2 shows structural similarity to clan CD proteases [4]. The mutation of a putative catalytic cysteine of RARP2 also prevented fragmentation of the TGN, but its proteolytic target or mode of action remain to be identified.

No change in the Golgi apparatus architecture was seen for *R*. *typhi* or *R*. *canadensis* that encode RARP2 with the same or greater number of ankyrin repeats as *R*. *rickettsii* Sheila Smith, and encode the putative catalytic cysteine site. Differences in the remaining coding sequence of RARP2 may influence the target affinity or function of these proteins.

The effects of RARP2 on Golgi architecture is apparent as early as 4 hr post-infection and dependent upon rickettsial translation but not *de novo* transcription. This effect is evident even at very low numbers of rickettsia/cell (<10) and suggests that RARP2 is extremely active or efficient. It may further indicate that RARP2 could be in low abundance which would be consistent with unsuccessful attempts to detect native RARP2 using antibody to recombinant protein [4]. RARP2 appears to be unstable though since inhibition of rickettsial protein synthesis by chloramphenicol led to recovery of TGN structure within 24 hr of treatment.

As previously shown, complementation of *R*. *rickettsii* Iowa with SS-RARP2 was not sufficient to restore virulence [4]. However, the Sheila Smith has 492 SNPs and 143 in/dels between it and the avirulent Iowa strain, thus it is likely that other factors also contribute to virulence [3,4].

A defect in glycosylation pattern of two cellular proteins was observed; the *trans*-Golgi protein, TGN46, and a plasma membrane localized protein complex, MHC-I. This effect was seen in cells infected with *R*. *rickettsii* Sheila Smith or *R*. *rickettsii* Iowa expressing SS-RARP2. It seems likely that the unknown cellular target of RARP2 is necessary for appropriate glycosylation and maturation of proteins trafficking through the Golgi apparatus. Appropriate glycosylation requires proper protein trafficking and sorting [35]. However, it cannot be presently determined whether the observed glycosylation defects are responsible for the disruption of TGN organization and inhibition of anterograde trafficking or a manifestation of the failure of de novo synthesized proteins to transit the disorganized Golgi apparatus.

Two independent methods demonstrated a general defect in delivery of plasma membrane proteins to the cell surface. There are multiple ways this may be of benefit to the rickettsiae. MHC-I dependent antigen presentation is critical for adaptive immune responses to many intracellular pathogens including rickettsiae [36,37]. Viruses have evolved multiple mechanisms to inhibit MHC class 1 antigen presentation. Viruses may specifically interfere with MHC-1 activity by blocking transcription of MHC-1 components, inhibition of peptide loading, retention of MHC-I in the ER, degradation of retained MHC-1 by proteosomes or lysosomes, and internalization of surface associated MHC-1, or in some cases, combinations of the above [38–41]. Some may more generally disrupt anterograde protein trafficking and thereby inhibit or slow MHC-1 trafficking to the cell surface [19]. An intracellular bacterium, *Orientia tsutsugamushi*, may also utilize specific and non-specific means to inhibit MHC-1 delivery to the cell surface [42]. Virulent *R*. *rickettsii* displays a dramatic and apparently global down-regulation of anterograde protein trafficking to the plasma membrane. It is likely that MHC-1 complexes loaded with rickettsial antigenic peptides are impaired in their delivery to the cell surface and may impede cytotoxic T-lymphocyte mediated cell lysis.

The general inhibition of trafficking to the plasma membrane may contribute to rickettsial pathogenesis in other ways. *Shigella* have been shown to disrupt E-cadherin trafficking to the plasma membrane to potentially disrupt epithelial layer coherence [21]. Spotted fever group rickettsiae primarily infect the vascular endothelium. Much of the pathogenesis of rickettsial infection is due to increased microvascular permeability [43]. Whether these effects might similarly be attributed to defects in trafficking of cellular adhesin molecules offer intriguing possibilities for future study.

## Materials and methods

### Cell lines and rickettsia

Vero76 cells (Cercopithecus aethiops; sex: unspecified) were grown in M199 medium plus 10% fetal bovine serum (FBS) at 37°C. *Rickettsia rickettsii* Sheila Smith (CP000848.1), Iowa (CP000766.3), Morgan(CP006010.1) or R (CP006009.1) were grown at 34°C in Vero76 cells in M199 medium plus 2% FBS. For purification of rickettsiae, Vero cells were lysed by Dounce homogenization followed by centrifugation through a 30% Renografin pad. Rickettsiae were washed twice in 250 mM sucrose, and either used directly for infections or stored in brain heart infusion at -80°C. Numbers of viable rickettsiae was determined by plaque assay on Vero cell monolayers.

### Immunofluorescence analysis and antibodies

Vero cells were grown on coverslips and infected at an MOI of 1. Cells were fixed at the indicated time points in 4% paraformaldehyde, permeabilized with 0.1% Triton X-100 in PBS for 5 min and stained with primary antibodies for an hour. Coverslips were washed with PBS and incubated with the respective secondary antibody diluted in PBS. The following commercial antibodies were used: anti-TGN46 (Cat#ab50595), anti-GM130 (Cat#ab52649), and anti-GFP (Cat#ab13920) from Abcam; and anti-Syntaxin 5 (Cat# sc365124) from Santa Cruz Biotechnology. Monoclonal antibody 13–2 against *R*. *rickettsii* rOmpB [44] was used for detection of rickettsia. Secondary F(ab')_2_-antibodies (DyLight_488, 594, 405_ or horseradish peroxidase conjugates) were from Jackson Immunoresearch. For lectin labeling, the Golgi apparatus was labeled with FITC-labeled *Helix pomatia* agglutinin (HPA) (1 μg/ml) (Invitrogen) or AlexaFluor_594_-wheat germ agglutinin (WGA) (2.5 μg/ml) (Invitrogen). Vital staining with C6-NBD-ceramide (Invitrogen) was performed as previously described [45]. Images were acquired on a Nikon Eclipse 80i microscope with a 60X 1.4-numerical aperture oil immersion objective and a Nikon DS-Qi1Mc camera. Confocal microscopy was performed on a Zeiss LSM-710. For quantification of cells showing labeling for GM130 or TGN46, images with at least 350 cells/replicate (n = 3) were acquired on a Cytation 5 Cell Imaging reader (BioTek Instruments).

#### Fluorescence in situ hybridization

In some experiments, rickettsiae were detected by Fluorescence in situ hybridization. For this, coverslips with fixed samples were incubated with the fluorescently labeled oligonucleotide probe Rick2287 (5’CCA ACC TGA GCT AAC CAT CG) in hybridization buffer (0.9 M NaCl, 20 mM TrisHCl (pH 8.0), 0.01% SDS, 35% formamide) overnight at 46°C [46]. Coverslips were washed with ice cold distilled water and processed further for immunofluorescence analysis as described above. Images were acquired on a Nikon Eclipse 80i microscope with a 60X 1.4-numerical aperture oil immersion objective and a Nikon DS-Qi1Mc camera. Confocal microscopy was performed on a Zeiss LSM-710. For quantification of cells showing labeling for GM130 or TGN46, images with at least 350 cells/replicate (n = 3) were acquired on a Cytation 5 Cell Imaging reader (BioTek Instruments).

### Ectopic expression of rickettsial proteins

pEGFP C1 encoding GFP-tagged RARP2 of Sheila Smith, Iowa or RARP2-C109A [4] were transfected into Hela cells using the Lipofectamine LTX Plus Kit (Invitrogen, LifeTechnologies) according to the manufacturer’s instructions. Cells were fixed 24 hours post transfection with paraformaldehyde and processed for immunofluorescence analysis as described above.

### Transmission electron microscopy

Vero cells were grown on Thermanox coverslips and infected with *R*. *rickettsii* Sheila Smith or *R*. *rickettsii* Iowa at an MOI of 1. Cells were rinsed with Hanks balanced salt solution, followed by fixation with PLP fixative (75 mM lysine, 37 mM sodium phosphate, 10 mM sodium periodate, 2% paraformaldehyde) plus 0.01% glutaraldehyde for 2 h at room temperature. Specimens were rinsed with PBS, permeabilized with 0.01% saponin in PBS for 5 minutes at room temperature and incubated with primary antibodies followed by incubation with peroxidase conjugated secondary antibody in 0.01% saponin in PBS. Samples were fixed for 1 h with 1.5% glutaraldehyde in 0.1 M sodium-cacodylate with 5% sucrose (pH 7.4), rinsed three times and developed using the Pierce diaminobenzidine (DAB) metal-enhanced substrate kit prior to embedding [47]. Samples were embedded in Spurr’s resin, and micrographs were acquired using a Hitachi 7500 transmission electron microscope (Hitachi High Technologies America, Inc.) at 80 kV and recorded on a bottom-mount AMT camera system (Advanced Microscopy Techniques Corp.)

### SDS-PAGE and immunoblotting

Lysates of Vero76 cells infected with rickettsiae were prepared by boiling of samples in Laemmli buffer for 10 min. Proteins were separated on 10% sodium dodecyl sulfate-polyacrylamide gels and transferred to a PVDF membrane at 100 V for 30 min. Membranes were incubated for 1 hr in Odyssey blocking buffer (LiCor), followed by incubation with primary antibody for 1 hr in Odyssey blocking buffer with 0.2% Tween-20. Blots were washed with Tris-buffered saline, 0.1% Tween-20 and incubated with appropriate secondary antibodies, followed by additional washing steps. Blots were imaged using the LI-COR Odyssey CLx Infrared Imaging System according to manufacturer's instructions.

### Deglycosylation assay

Vero76 cells were infected with rickettsiae at an MOI of 1 and lysed in deionized water at 48 hpi. Lysates were processed and treated with Protein Deglycosylation mix II (NEB) according to the manufacturer’s instructions. Laemmli sample buffer was added to an equal volume and heated to 99°C for 10 min and analyzed by immunoblot analysis. Untreated samples without the addition of enzyme were used as controls.

### Monitoring of protein trafficking using the RUSH system

Str-KDEL_TNF-SBP-EGFP (Addgene plasmid # 65278) and Str-KDEL_SBP-EGFP-Ecadherin (Addgene plasmid # 65286) were a gift from Franck Perez [9]. Vero76 cells were transfected with different RUSH-constructs using the Lipofectamine LTX Plus Kit (Invitrogen, LifeTechnologies) according to the manufacturer’s instructions. Medium was exchanged four hours after transfections for medium supplemented with 50 ng/ml avidin. 24 hours after transfections, transfected cells were infected with rickettsiae at an MOI of 1 using media supplemented with avidin. Trafficking of reporter proteins was induced by exchanging the medium for medium containing 40 μM biotin. Cells were fixed 80 min after addition of biotin with 4% paraformaldehyde but not permeabilized. Fixed but unpermeabilized cells were incubated with a chicken anti-GFP antibody (1:2,000) followed by anti-chicken-Ig-AlexaFluor_568_ secondary antibody (1:1,000, Abcam) to detect surface exposed GFP. Susequently, cells were permeabilized with PBS plus 0.1% Triton X-100 and rickettsiae were detected with a rickettsia-specific monoclonal antibody and anti-mouse-Ig-AlexaFluor_488_ secondary antibody. Experiments were performed in biological triplicates.

### Pulse-labelling and pulldown of eukaryotic surface proteins

Vero76 cells were infected with rickettsiae at an MOI of 1. Medium was exchanged 24 hpi for prewarmed medium with 100 μCi ^35^S for 15 min. Wells were rinsed with HBSS, followed by addition of pre-warmed M199 medium with 10% FBS. After 2 hours, wells were rinsed three times with HBSS and sulfo-NHS biotin (1mg/ml) in PBS was added for 30 min to label surface proteins. Cells were rinsed three times with PBS with 100 mM glycine and lysed in RIPA buffer (150 mM NaCl, 1% NP-40, 0.5% Deoxycholic Acid, 0.1% SDS, 50 mM Tris-HCl, pH 8.0, plus 1 mM PMSF). Samples were pre-cleared by centrifugation (5,000 x g for 5 min) and incubated with 100 μl NeutrAvidin resin (Pierce) for 30 min at room temperature. Samples were pelleted by brief centrifugation, washed 3 times with PBS, and solubilized in Laemmli sample buffer for SDS-PAGE.

Alternatively, cultures were ^35^S-labeled as above and lysed in RIPA-buffer and an aliquot saved for SDS-PAGE. The remainder of the sample was pre-cleared by centrifugation and incubated for 1 hr at room temperature with anti-HLA A,B,C antibody (Epitomics, clone w6/32). 50 μl of Protein G Plus-agarose suspension (Millipore) was added and incubation continued for an addition 1 hr at room temperature. Samples were pelleted by brief centrifugation, washed 3 times with PBS, and resuspended in 50 mM NaCitrate, pH 5.5. Samples were then split. To one half was added 125 U EndoH (NEB) and the other kept as an untreated control. Samples were incubated overnight at 37°C, pelleted by brief centrifugation, and solubilized in Laeemli buffer for SDS-PAGE and immunoblotting against anti-HLA class 1 A,B,C (Abcam).

## Supporting information

S1 Fig Infection with *R*. *rickettsii* Sheila Smith causes dispersal of the *trans*-Golgi. Cells infected with *R*. *rickettsii* Sheila Smith (SS) (green), R. rickettsii Iowa (Iowa) (green) and uninfected controls (Un) were stained for the Golgi tethering protein Giantin (red); and the *trans*-Golgi proteins Golgin-97, GCC1 and GCC2 (red). Nucleic acids were stained with DAPI (blue). Bar = 10 um.
(TIF)

S2 Fig Immunohistochemistry of Vero cells infected and as prepared for immunoelectron microscopy. Vero cells were infected with *R*. *rickettsii* Sheila Smith or R. rickettsii Iowa at an MOI of 1 or uninfected control cells (Un) and fixed at 48 hpi. Primary antibodies targeted GM130 or TGN46, followed by horseradish peroxidase conjugated secondary antibodies and diaminobenzidine-based detection. Bar = 10 um.
(TIF)

S3 Fig Fragmentation of the TGN correlates with RARP2 structure in strains of *R*. *rickettsii* differing in virulence. The *trans*-Golgi network protein TGN46 is dispersed in cells infected with the virulent strains Sheila Smith (SS) and Morgan (Mor) that express a full-length RARP2, but not in uninfected cells or cells infected with the avirulent Iowa strain (Iowa) or the moderately virulent R strain (R) which express a truncated version of RARP2. Vero cells were infected at an MOI of 1 and fixed 48 hpi. Unifected cells (Un) served as a negative control. The *cis*-Golgi protein GM130 (red) and the *trans*-Golgi protein TGN46 (green) were stained with specific antibodies. Nucleic acids were stained with DAPI (blue). Bar = 10 μm.
(TIF)

S4 Fig Effect of typhus group rickettsiae on Golgi morphology. Vero cells were infected with *R*. *typhi* or *R*. *canadensis* at and MOI of 1 and incubated for 48 hr at 34°C before fixation with 100% methanol. Cultures were labeled with monoclonal antibody 13–6 (*R*. *canadensis*) or a rabbit anti-*R*. *prowazekii* polyclonal serum (*R*. *typhi*) (green) and anti-TGN46 (Abcam) or (InVitrogen) antibody, respectively (red). Nuclei are counterstained with DAPI (blue). Bar = 10 μm.
(TIF)

S5 Fig Expression of SS-RARP2 by *R*. *montanensis* causes dispersal of the *trans*-Golgi network. The vector pRAMF2 encoding for SS-RARP2 or Io-RARP2 was transformed into *R*. *montanensis*, a species that does not encode a homologue of RARP2, and used to infect Vero cells. Cells were infected with *R*. *montanensis* expressing SS-RARP2 (Rmont SS-RARP2,) expressing Io-RARP2 (Rmont Io-RARP2). Cells were fixed 24 hpi and the *trans*-Golgi protein TGN46 (red) was detected using a specific antibody. GFP-expressing rickettsiae are shown in green, nucleic acids stained with DAPI in blue. Bar = 10μm.
(TIF)

S6 Fig Higher magnification and quantitation of ectopic expression of SS-RARP2, Io-RARP2, and SS-RARP2-C109A. A) Ectopic expression of SS-RARP2 causes dispersal of the *trans*-Golgi network. Sheila Smith-RARP2 (SS), Iowa RARP2 (Iowa), or the SS-RARP2-C109A mutant (C109A) were expressed in Vero cells as EGFP fusions from pEGFP-C1 and probed for TGN46 (red). The TGN is dispersed in cells expressing SS-RARP2 GFP (green), but not in cells expressing Iowa-RARP2 GFP (green) or negative control cells expressing EGFP (GFP). Ectopically expressed SS-RARP2-GFP and SS-RARP2-C109A are enriched in vesicular structures as previously observed [4]. Outlines of transfected cells expressing SS-RARP2 are shown in white. Bar = 10 μm. B) Quantitation of the TGN46 dispersal in cells ectopically expressing Sheila Smith-RARP2 (SS), Iowa RARP2 (Iowa), SS-RARP2-C109A mutant (C109A), or negative control cells expressing EGFP (GFP). TGN46 localization was determined for between 24–51 transfected cells per construct in two technical replicates each from two biological replicates and shown as percent cells with dispersed TGN. Shown is the mean +/- the S.E.M. Statistics were performed using an unpaired Student's t-test. Significant differences between SS-RARP2 and, Iowa-RARP2, SS-RARP2-C109A, or GFP control are indicated (**** p<0.0001) The difference between Iowa-RARP and SS-RARP2-C109A or GFP control was not signficant (ns).
(TIF)

S7 Fig RARP2 mediated TGN dispersal is reversible. Chloramphenicol was added to *R*. *rickettsii* Sheila Smith-infected cells at 24 hpi to inhibit bacterial protein synthesis and then incubated and additional 24 hr. Addition of chloramphenicol resulted in restoration of Golgi morphology by 48 hpi. TGN46 (red); GM130 (green); rickettsiae (white); and DAPI (blue. Bar = 10 μm.
(TIF)

S8 Fig An intact *cis*-Golgi apparatus is not required for *R*. *rickettsii* replication. Vero cells with infected with *R*. *rickettsii* Sheila Smith or Iowa and Brefeldin A added to 1 μg/ml at 6 hpi. Infected cells were lysed and replated for PFUs at 24 and 48 hpi. No difference in growth rate was observed for either strain. Mean +/- SE; N = 3.
(TIF)
