# Supplementary figures and images for: Selective fragmentation of the trans-Golgi apparatus by Rickettsia rickettsii
Source: PLoS Pathog. 2020 May 18;16(5):e1008582. doi: 10.1371/journal.ppat.1008582 (PMC7259798; doi:10.1371/journal.ppat.1008582)

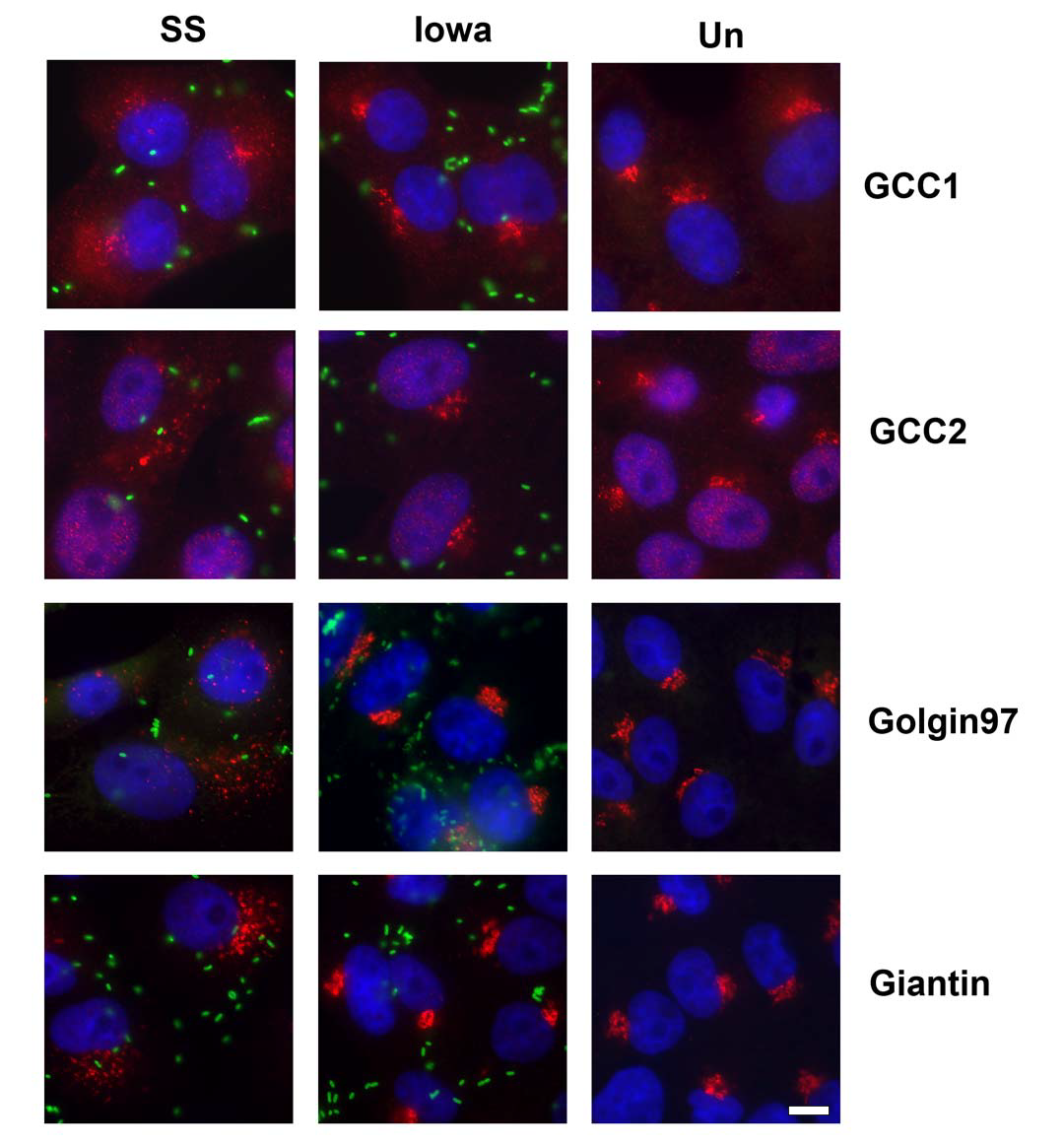

Supplement: S1 Fig — Cells infected with R. rickettsii Sheila Smith (SS) (green), R. rickettsii Iowa (Iowa) (green) and uninfected controls (Un) were stained for the Golgi tethering protein Giantin (red); and the trans-Golgi proteins Golgin-97, GCC1 and GCC2 (red). Nucleic acids were stained with DAPI (blue). Bar = 10 um. (TIF) [file ppat.1008582.s001.tif]

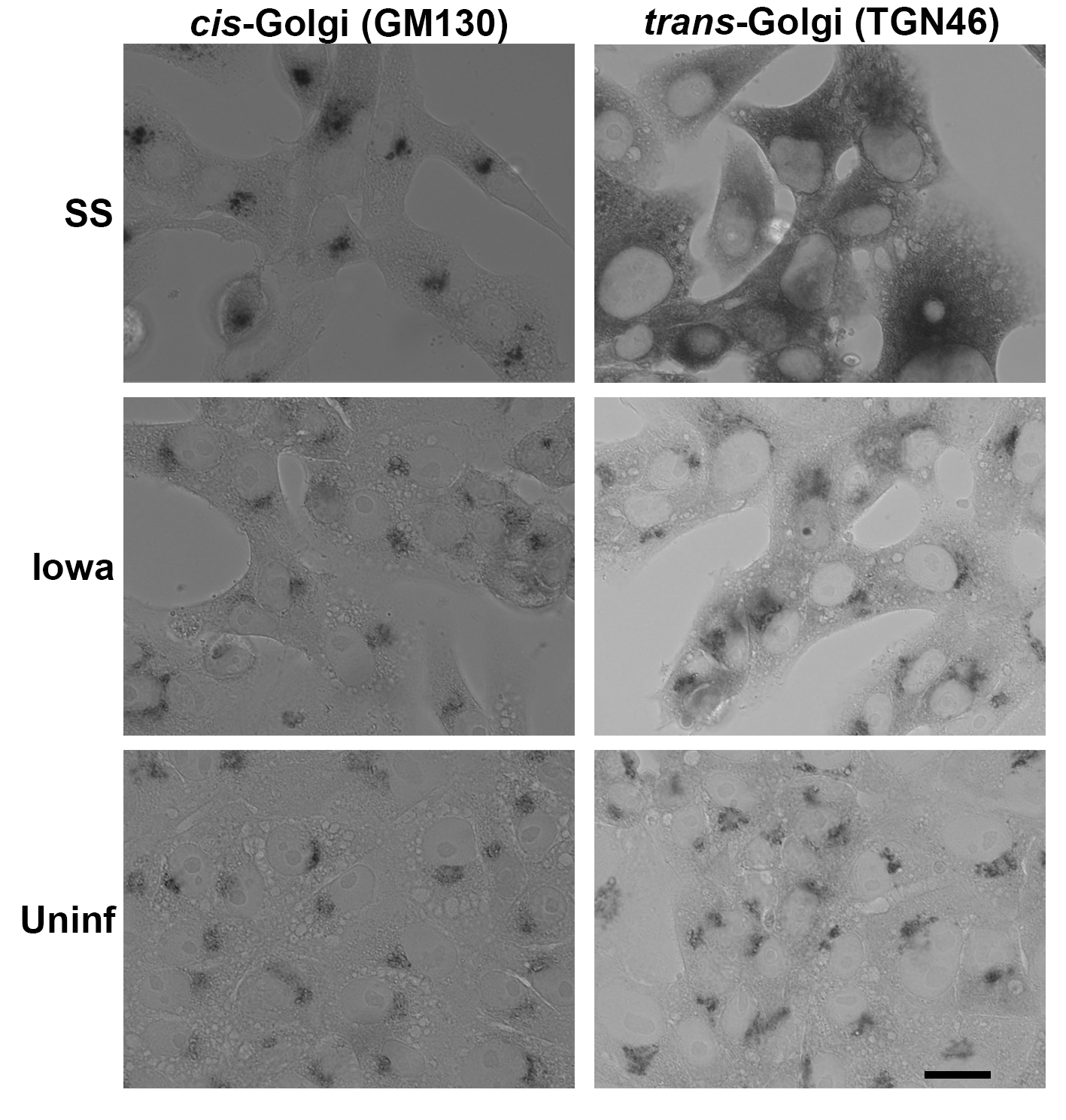

Supplement: S2 Fig — Vero cells were infected with R. rickettsii Sheila Smith or R. rickettsii Iowa at an MOI of 1 or uninfected control cells (Un) and fixed at 48 hpi. Primary antibodies targeted GM130 or TGN46, followed by horseradish peroxidase conjugated secondary antibodies and diaminobenzidine-based detection. Bar = 10 um. (TIF) [file ppat.1008582.s002.tif]

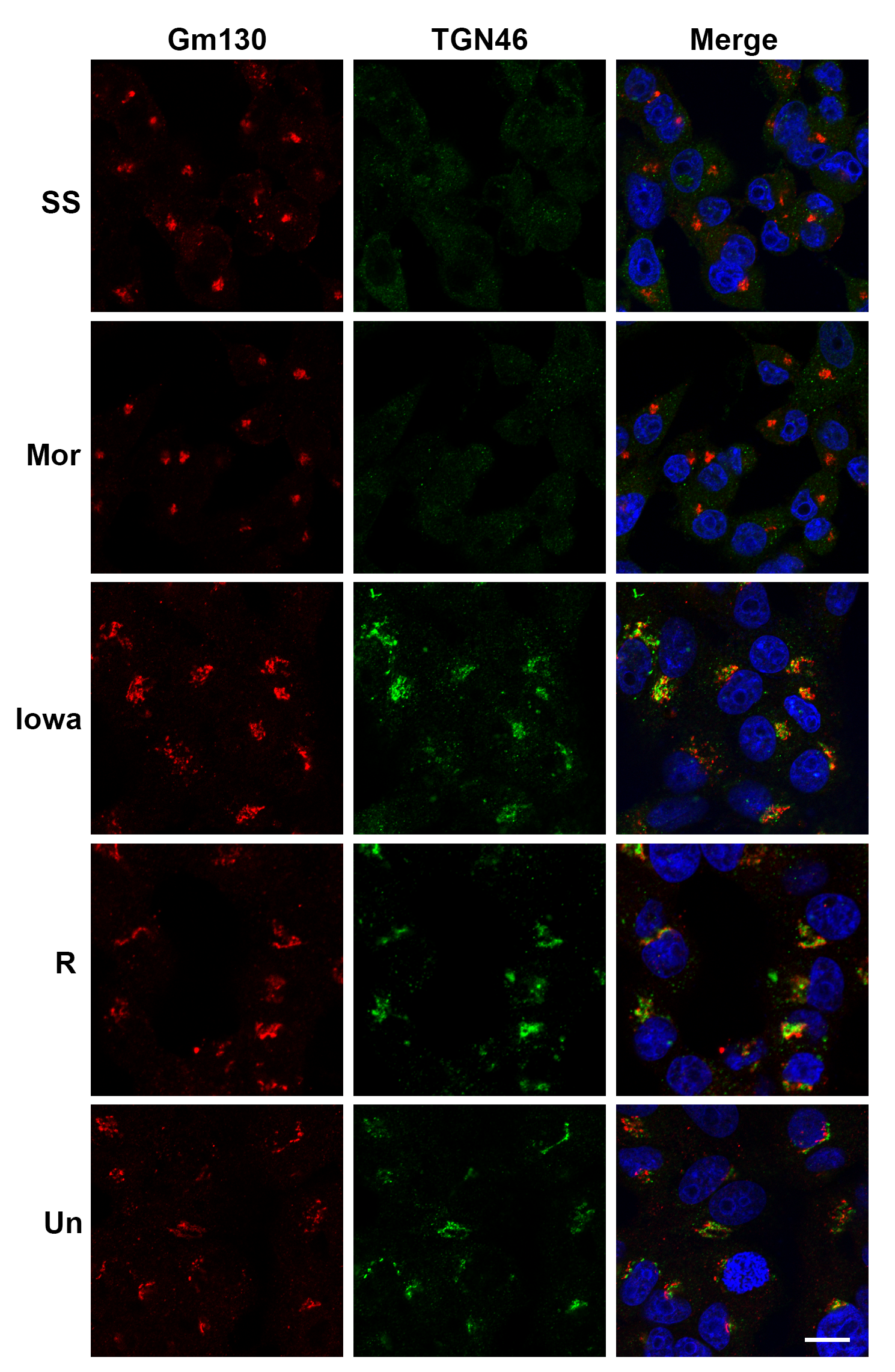

Supplement: S3 Fig — The trans-Golgi network protein TGN46 is dispersed in cells infected with the virulent strains Sheila Smith (SS) and Morgan (Mor) that express a full-length RARP2, but not in uninfected cells or cells infected with the avirulent Iowa strain (Iowa) or the moderately virulent R strain (R) which express a truncated version of RARP2. Vero cells were infected at an MOI of 1 and fixed 48 hpi. Unifected cells (Un) served as a negative control. The cis-Golgi protein GM130 (red) and the trans-Golgi protein TGN46 (green) were stained with specific antibodies. Nucleic acids were stained with DAPI (blue). Bar = 10 μm. (TIF) [file ppat.1008582.s003.tif]

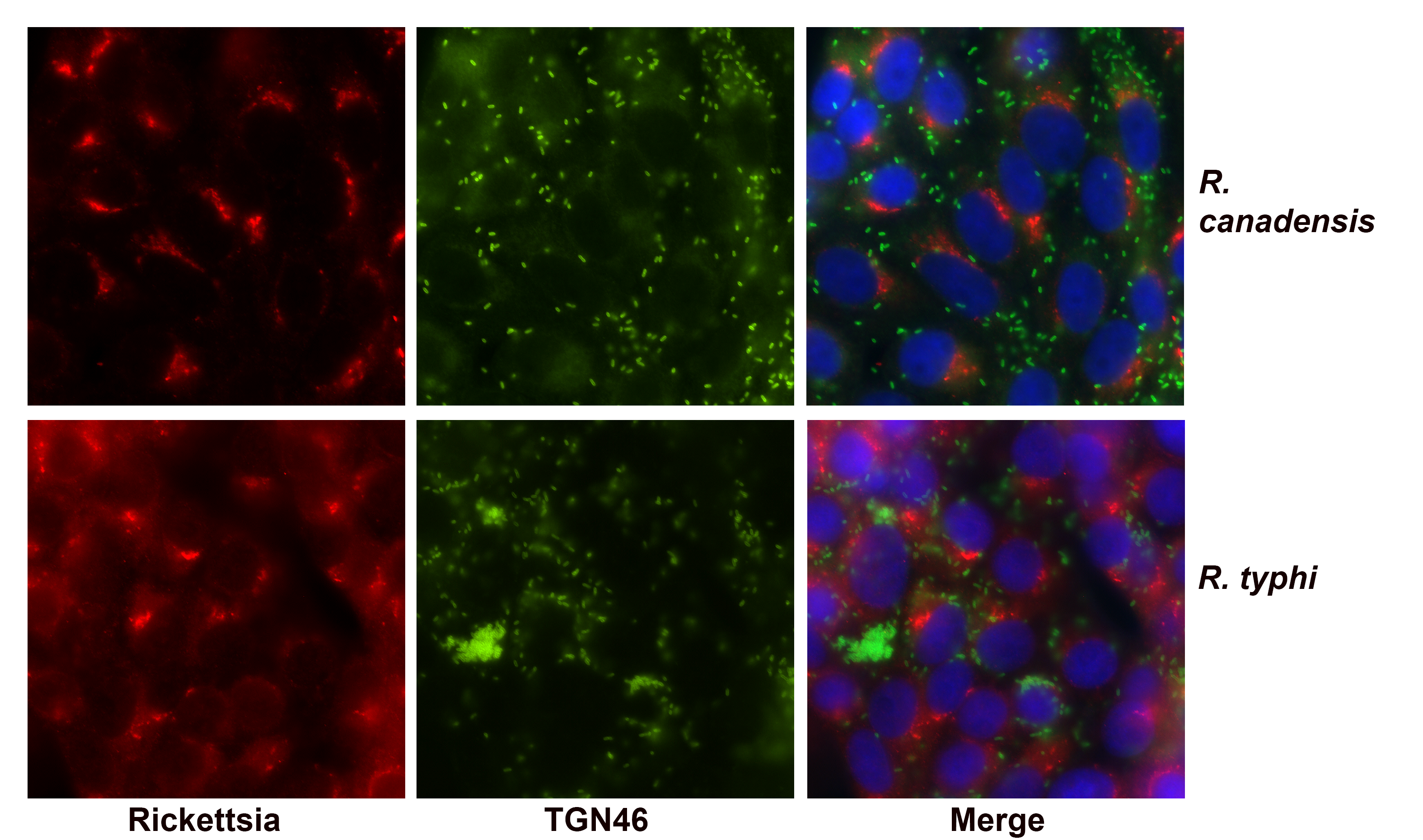

Supplement: S4 Fig — Vero cells were infected with R. typhi or R. canadensis at and MOI of 1 and incubated for 48 hr at 34°C before fixation with 100% methanol. Cultures were labeled with monoclonal antibody 13–6 (R. canadensis) or a rabbit anti-R. prowazekii polyclonal serum (R. typhi) (green) and anti-TGN46 (Abcam) or (InVitrogen) antibody, respectively (red). Nuclei are counterstained with DAPI (blue). Bar = 10 μm. (TIF) [file ppat.1008582.s004.tif]

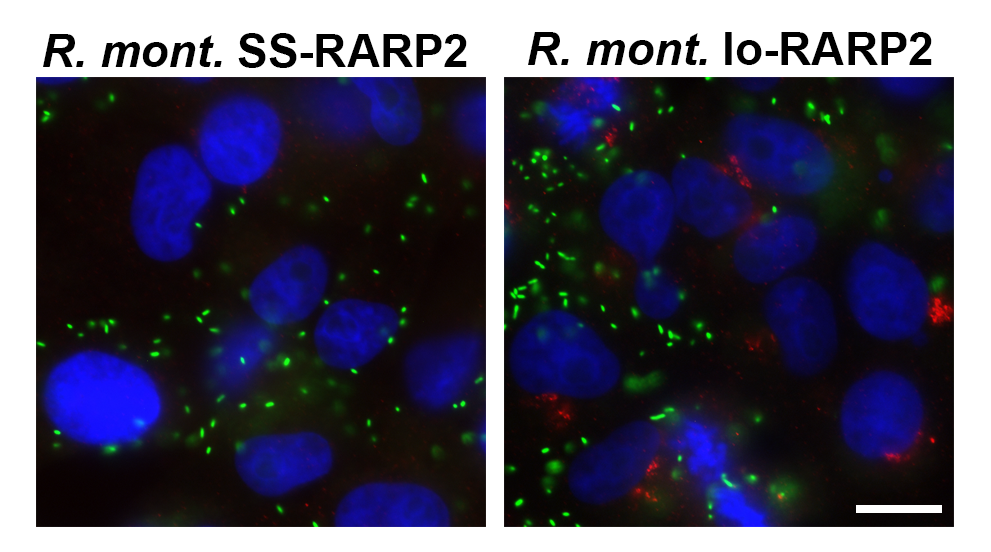

Supplement: S5 Fig — The vector pRAMF2 encoding for SS-RARP2 or Io-RARP2 was transformed into R. montanensis, a species that does not encode a homologue of RARP2, and used to infect Vero cells. Cells were infected with R. montanensis expressing SS-RARP2 (Rmont SS-RARP2,) expressing Io-RARP2 (Rmont Io-RARP2). Cells were fixed 24 hpi and the trans-Golgi protein TGN46 (red) was detected using a specific antibody. GFP-expressing rickettsiae are shown in green, nucleic acids stained with DAPI in blue. Bar = 10μm. (TIF) [file ppat.1008582.s005.tif]

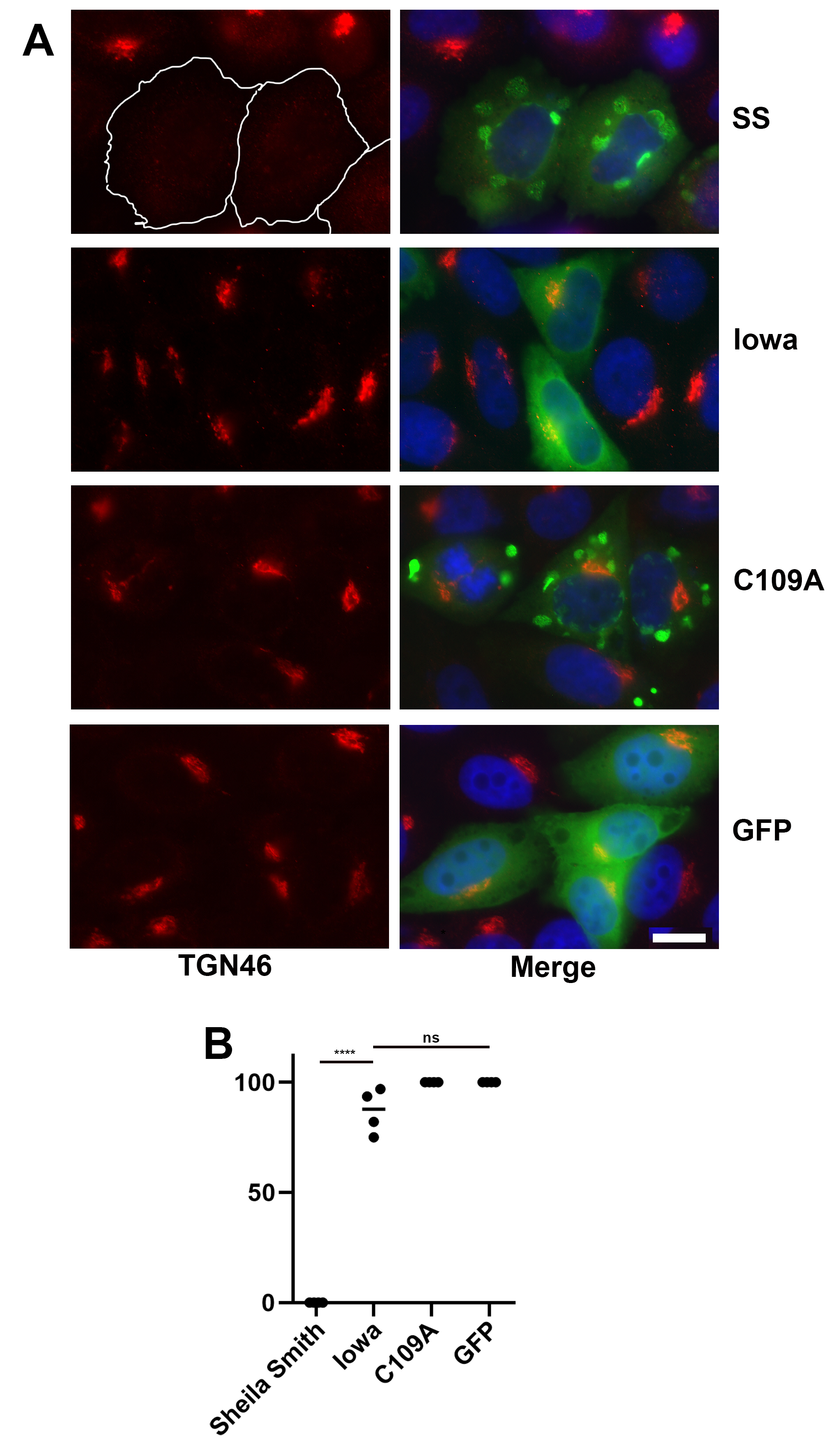

Supplement: S6 Fig — A) Ectopic expression of SS-RARP2 causes dispersal of the trans-Golgi network. Sheila Smith-RARP2 (SS), Iowa RARP2 (Iowa), or the SS-RARP2-C109A mutant (C109A) were expressed in Vero cells as EGFP fusions from pEGFP-C1 and probed for TGN46 (red). The TGN is dispersed in cells expressing SS-RARP2 GFP (green), but not in cells expressing Iowa-RARP2 GFP (green) or negative control cells expressing EGFP (GFP). Ectopically expressed SS-RARP2-GFP and SS-RARP2-C109A are enriched in vesicular structures as previously observed [4]. Outlines of transfected cells expressing SS-RARP2 are shown in white. Bar = 10 μm. B) Quantitation of the TGN46 dispersal in cells ectopically expressing Sheila Smith-RARP2 (SS), Iowa RARP2 (Iowa), SS-RARP2-C109A mutant (C109A), or negative control cells expressing EGFP (GFP). TGN46 localization was determined for between 24–51 transfected cells per construct in two technical replicates each from two biological replicates and shown as percent cells with dispersed TGN. Shown is the mean +/- the S.E.M. Statistics were performed using an unpaired Student's t-test. Significant differences between SS-RARP2 and, Iowa-RARP2, SS-RARP2-C109A, or GFP control are indicated (**** p<0.0001) The difference between Iowa-RARP and SS-RARP2-C109A or GFP control was not signficant (ns). (TIF) [file ppat.1008582.s006.tif]

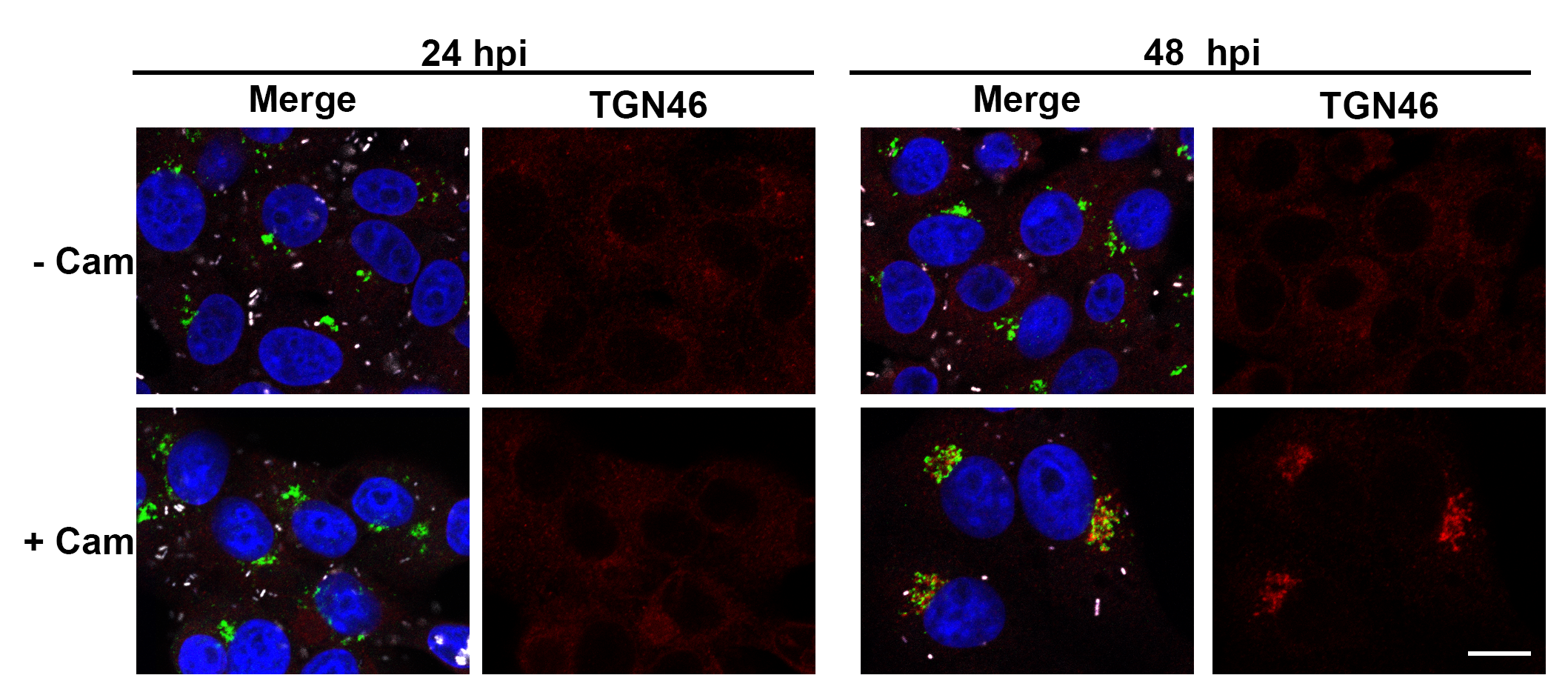

Supplement: S7 Fig — Chloramphenicol was added to R. rickettsii Sheila Smith-infected cells at 24 hpi to inhibit bacterial protein synthesis and then incubated and additional 24 hr. Addition of chloramphenicol resulted in restoration of Golgi morphology by 48 hpi. TGN46 (red); GM130 (green); rickettsiae (white); and DAPI (blue. Bar = 10 μm. (TIF) [file ppat.1008582.s007.tif]

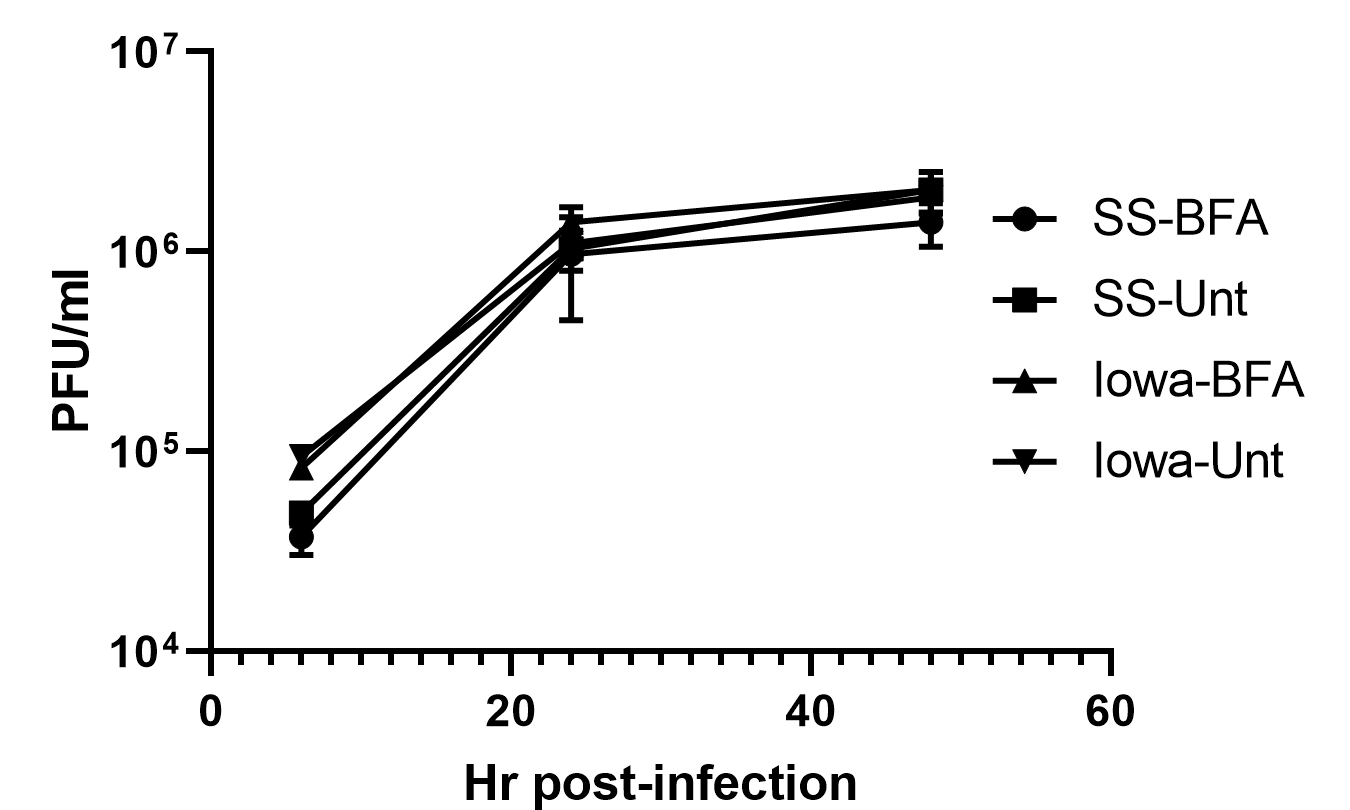

Supplement: S8 Fig — Vero cells with infected with R. rickettsii Sheila Smith or Iowa and Brefeldin A added to 1 μg/ml at 6 hpi. Infected cells were lysed and replated for PFUs at 24 and 48 hpi. No difference in growth rate was observed for either strain. Mean +/- SE; N = 3. (TIF) [file ppat.1008582.s008.tif]
